# Modular engineering of thermoresponsive allosteric proteins

**DOI:** 10.1038/s41589-026-02151-y

**Published:** 2026-02-12

**Authors:** Ann-Sophie Kroell, Kira H. Hoffmann, Nikolas A. Motzkus, Nina Lemmen, Nele Happ, Benedict Wolf, Anna-Lisa von Bachmann, Nicholas Southern, Felicitas Vogd, Sabine Aschenbrenner, Dominik Niopek, Jan Mathony

**Affiliations:** 1 Institute of Pharmacy and Molecular Biotechnology (IPMB), Faculty of Engineering Sciences, Heidelberg University, Heidelberg, Germany; 2 Zuse School ELIZA, Darmstadt, Germany

**Keywords:** Synthetic biology, Transcription, Protein design

## Abstract

Thermogenetics enables noninvasive spatiotemporal control over protein activity in living cells and tissues, yet its applications have largely been restricted to transcriptional regulation and membrane recruitment. Here, we present a generalizable strategy for engineering thermosensitive allosteric proteins through the insertion of optimized *Avena* *sativa* LOV2 domain variants. Applying this approach to a diverse set of structurally and functionally unrelated proteins in *Escherichia* *coli*, we generated potent, thermoswitchable chimeric variants that can be tightly controlled within narrow temperature ranges (37–41 °C). Extending this strategy to mammalian systems, we engineered CRISPR–Cas genome editors directly modulated by subtle temperature changes within the physiological range. Lastly, we showcase the incorporation of a chemoreceptor domain as an alternative thermosensing module, suggesting thermosensitivity to be a widespread feature in receptor domains. This work expands the toolkit of thermogenetics, providing a blueprint for temperature-dependent control of virtually any protein of interest.

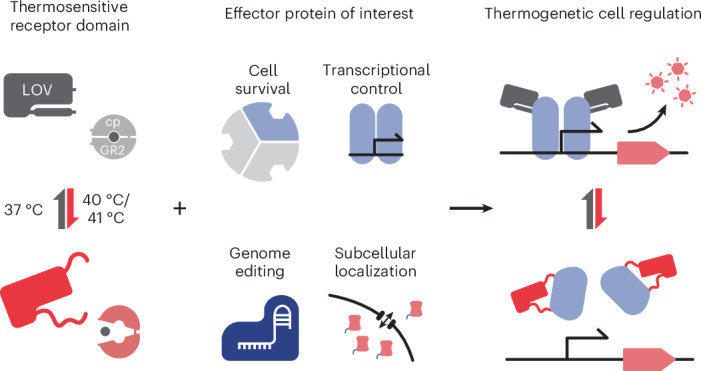

## Abstract (web summary)

Thermogenetics enables spatiotemporal control of protein activity using temperature. Now, engineering of a compact, insertable thermoresponsive protein module diversifies the classes of proteins amenable to allosteric thermoregulation.

## Main

Engineered switchable proteins are widely applied in basic research and biotechnology. They are typically based on naturally occurring proteins that are modified to be activated or deactivated by exogenous stimuli, most commonly light or chemicals, allowing researchers to remotely control various cellular processes. With respect to future biomedical applications, temperature represents a particularly attractive physical cue because of the increased tissue penetration compared to light and the superior spatiotemporal precision compared to chemicals^1–3^. Efficient approaches for engineering thermoresponsive proteins are lacking, however, and the few existing strategies are mostly limited to the control of gene expression^4–6^. Recent work used the *Botrytis* *cinerea* LOV4 (*Bc*LOV4) domain for temperature-dependent recruitment of proteins to the plasma membrane^7,8^. While this method is generally compatible with various different effector proteins, it relies on a large protein domain (*Bc*LOV4 is approximately 600 amino acids) and is restricted to effectors, the activity of which depends on plasma membrane localization.

To address these key limitations, we aimed to develop a generalizable approach for thermogenetic control through modular engineering of temperature-dependent protein allostery. By inserting the *Avena* *sativa* LOV2 (*As*LOV2) domain from phototropin 1 and improved mutants thereof into effector proteins, we demonstrate the modular engineering of thermoresponsive hybrids. Starting with the bacterial transcription factor AraC as a proof of concept, we achieved potent and tunable activity switching in response to small temperature shifts between 37 °C and 41 °C. We successfully transferred this concept to several additional proteins, including different CRISPR–Cas9 gene editors and transactivators, and showcase the broad applicability of the concept in bacteria and in mammalian cells. Lastly, we extend the method to a second receptor using a glucocorticoid ligand-binding domain and demonstrate the dual regulation of genome editing by the US Food and Drug Administration (FDA)-approved drug cortisol and temperature. Our work overcomes a major bottleneck in thermogenetics by introducing a generalizable approach for engineering thermogenetic switches that can be controlled within the narrow human physiological temperature range and are not limited to specific applications or protein classes.

## Results

### Engineering a thermoswitchable AraC variant by *As*LOV2 insertion

**Fig. 1:**
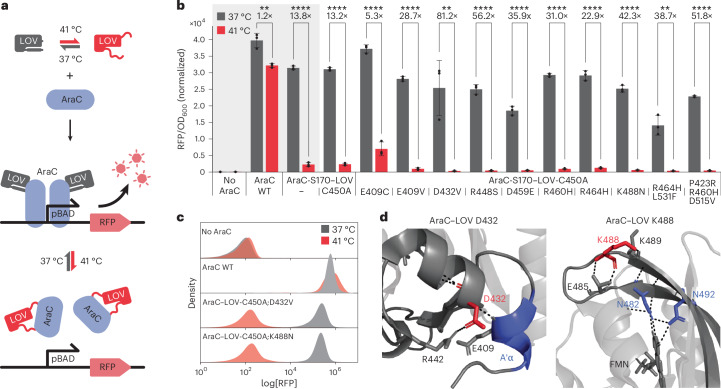
Engineering a thermoswitchable *E*. *coli* transcription factor by *As*LOV2 domain insertion. **a**, Assay schematics. **b**,**c**, *E*. *coli* transformed with plasmids encoding the indicated AraC–LOV variants and an mRFP reporter were induced with 400 µM IPTG and 16 mM arabinose and incubated at 37 °C or 41 °C for 16 h followed by measurements of reporter fluorescence (RFP) and culture density (OD_600_) in a plate reader (**b**) or flow cytometry (**c**). **d**, Close-up views of the *As*LOV2 crystal structure (PDB 2V0U). Substituted residues are marked in red and functionally critical elements are marked in blue. Hydrogen bonds are indicated. In **b**, data represent the mean ± s.d. (*n* = 3 independent experiments). Statistical analysis was conducted using a two-sided Student’s *t*-test. **P < 0.01 and ****P < 0.0001. WT, wild-type. Source Data

An ideal thermoresponsive module would comprise a compact protein domain that exerts a large conformational rearrangement in response to small changes in temperature. Insertion of such a domain into an effector protein of choice could, thus, couple the activity of the effector to the conformational state of the thermosensor. Intrigued by the relatively low stability of many LOV domains^7,9,10^, we decided to test the widely applied *As*LOV2 for its response to temperature changes. *As*LOV2 naturally acts as a photoreceptor, responding to blue-light excitation (450 nm) with conformational changes that result in the reversible undocking of its N-terminal and C-terminal A′α and Jα helices, respectively, from the core^11,12^. Inserting *As*LOV2 into effector proteins is a common strategy for engineering light-responsive proteins by reversibly perturbing the effector’s structural integrity upon *As*LOV2 photoexcitation^13^. To assess whether the same effect can be achieved in the dark by thermal stimulation alone, we used an optogenetic variant of the transcription factor AraC carrying an *As*LOV2 insertion after residue S170, which we previously established for coregulating transgene expression by light and arabinose in *E*. *coli*^14^ (Fig. 1a). At 37 °C, both wild-type AraC and the AraC–LOV domain hybrid mediated strong expression of a pBAD-driven, monomeric red fluorescent protein (RFP) (Supplementary Fig. 1). At 41 °C, however, strongly reduced RFP expression was consistently observed for AraC–LOV, whereas the wild-type AraC control remained unaffected, indicating a potent thermoresponse of AraC–LOV (Fig. 1b and Supplementary Fig. 1). Importantly, cell growth was only slightly affected by the elevated temperature (Supplementary Fig. 1a). Furthermore, substitution of the *As*LOV2 C450 residue, which is central to the native *As*LOV2 photocycle, to alanine did not affect the temperature switching of the AraC–LOV hybrid and was included in all downstream constructs to avoid cross-activation during short exposure to ambient room light directly before measurements.

To optimize the dynamic range of thermal control, we performed error-prone PCR on LOV-C450A and screened the resulting library of *E*. *coli* (complexity: ~5 × 10^6^) for temperature-dependent reporter expression on agar plates (Methods and Supplementary Fig. 2). We identified several *As*LOV2 point mutants, including D432V, R448S, K488N and P423R/R460H/D515V, that showed up to 82-fold reduction in activity upon heat induction compared to only a 13.2-fold change observed for the parental hybrid protein (Fig. 1b,c and Supplementary Fig. 3). This improvement was primarily caused by a strongly reduced reporter expression in the 41 °C off state, as indicated by plate reader measurements and flow cytometry. To investigate potential temperature effects on protein stability, we performed western blots of wild-type AraC, AraC–LOV-C450A and two mutants (E409C and R464H/L531F) that showed unexpected bimodal distributions in the cytometry assay. The results revealed strongly reduced protein levels of the AraC–LOV variants compared to wild-type AraC under both conditions (Supplementary Fig. 4). In contrast, increased temperature had no significant effect on the protein levels, suggesting that the observed activity changes were not caused by temperature-dependent protein degradation.

**Extended Data Fig. 1:**
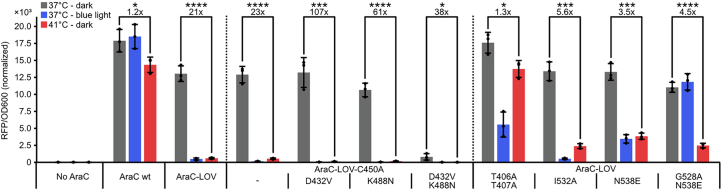
Improved AraC-LOV variants respond similarly to the light and temperature stimuli whereas *As*LOV2 stabilizing mutations exhibit stimulus-specific effects. *E. coli* carrying a pBAD-mRFP reporter and expressing the indicated AraC variant or a dummy control protein were incubated for 16 h at the indicated light and temperature conditions. RFP expression (normalized to OD) was assessed in a plate reader. The dotted line separates the previously reported stabilizing mutations. Data represent the mean +/- SD, n = 3 independent experiments. Fold changes are indicated. *P < 0.05, ***P < 0.001, ****P < 0.0001, two-sided Student’s t-test. wt, wild-type. Source Data

From the best-performing AraC–LOV hybrids, we selected the mutants D432V and K488N for further characterization. Both substitutions are likely to have destabilizing effects. D432 forms a hydrogen bond with E409 in the mechanistically important A′α helix (Fig. 1d), while K488 stabilizes a loop connecting two β-sheets, both in direct contact with the flavin mononucleotide chromophore (Fig. 1d). Consistent with this hypothesis, a D432V/K488N double mutant resulted in strongly reduced activity already at 37 °C, indicating that these substitutions synergistically impair stability (Extended Data Fig. 1). When comparing temperature response to blue-light-induced transcriptional deactivation, we found that all AraC–LOV hybrids were inactive both at 41 °C in the dark and at 37 °C under blue-light exposure, in line with previous reports on the LOV-C450A mutant acquiring a constitutive lit state during prolonged illumination^15,16^ (Extended Data Fig. 1). To further investigate the similarity between heat-induced and light-induced deactivation, we compared multiple A′α and Jα helix-stabilizing substitutions that qualitatively reduced the responsiveness to both stimuli^17,18^. Notably, the LOV-T406A/T407A double mutant showed a markedly stronger loss of thermal than light responsiveness whereas LOV-G528A/N538E displayed the opposite trend (Extended Data Fig. 1). These findings indicate that helix undocking in response to light versus temperature may not necessarily proceed through the identical molecular mechanism.

**Fig. 2:**
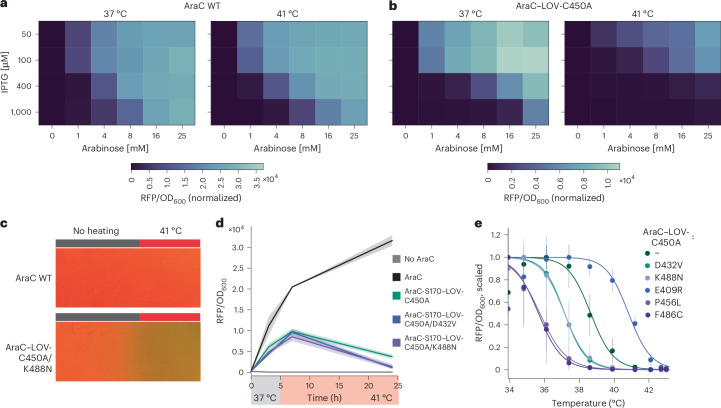
Thermosensitive AraC–LOV hybrids are precisely tunable and enable spatiotemporal control of gene expression. **a**,**b**, *E*. *coli* carrying a pBAD-mRFP reporter and wild-type AraC (**a**) or AraC–LOV-C450A (**b**) were incubated for 16 h at 37 °C or 41 °C in the presence of inducers at the indicated concentration. Expression of the AraC variants is IPTG inducible, while AraC activity is arabinose dependent. RFP fluorescence and OD_600_ were evaluated in a plate reader. **c**, Photographs of spatial gene expression control. AraC and AraC–LOV reporter strains were plated on agar and one half of the tray was heated while the other half was kept at room temperature for 16 h. **d**, *E*. *coli* containing a pBAD-mRFP reporter and expressing the indicated AraC variant or a dummy control protein were incubated at 37 °C. After 5 h, the temperature was increased to 41 °C followed by another 19 h of incubation. OD_600_ and RFP expression (normalized to OD) were periodically assessed in a plate reader. **e**, Temperature response profiles of AraC–LOV variants. Samples were prepared as in **a**, but incubated for 16 h at different temperatures. Data were min–max-normalized for each sample (raw data in Supplementary Fig. 7). **d**,**e**, Data represent the mean ± s.d. (*n* = 3 independent experiments). Source Data

Continuing with a detailed characterization of our thermogenetic system, inducer dose escalation experiments revealed that reporter activation by AraC–LOV-C450A at 37 °C and its thermal response at 41 °C are expression dependent and tunable over a wide range of concentrations (Fig. 2a,b and Supplementary Fig. 5). However, very high inducer levels (that is, 1,000 µM IPTG and certain conditions with 25 mM arabinose) resulted in compromised thermal switching, likely because of AraC accumulation in the cells. For all subsequent experiments, we chose the 400 µM IPTG and 16 mM arabinose condition, consistent with our previous work on optogenetic AraC variants^14^. Next, focusing on our lead candidates, we showcased efficient spatial control when *E*. *coli* expressing AraC–LOV-C450A/K488N were grown on agar plates and a heat gradient was applied (Fig. 2c). Moreover, we demonstrated reversible temperature control through timed incubation of liquid cultures at different temperatures for both the D432V and the K488N mutant (Fig. 2d and Supplementary Fig. 6). Subsequent experiments, in which identical samples were incubated at several temperatures between 34 and 43 °C in parallel, revealed a sharp decrease in transcriptional activation within a range of only 3–4 °C for the improved AraC–LOV hybrids (Fig. 2e and Supplementary Fig. 7). Using our screening pipeline, we further identified three single-point substitutions, E409R, P456L and F486C, with shifted transition temperatures in the range of 34–37 °C, which allowed even cold-induced protein activation at temperatures below 37 °C (Fig. 2e and Supplementary Fig. 7).

### Allosteric thermocontrol of diverse bacterial protein classes

**Fig. 3:**
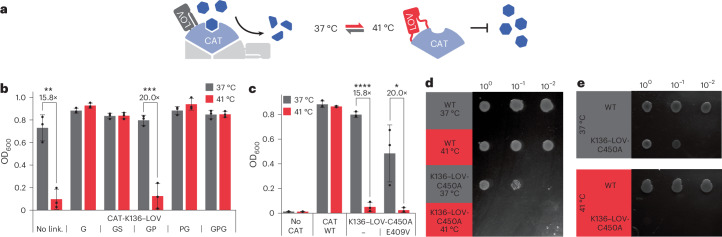
An engineered CAT–LOV hybrid acts as a heat-inducible microbial kill switch. **a**, Assay schematics. **b**,**c**, *E*. *coli* cultures expressing either wild-type CAT, no CAT, the indicated CAT-K136–LOV linker variants (**b**) or CAT-K136–LOV variants with point substitutions in the LOV domain (**c**) were grown in the presence of chloramphenicol. Samples were incubated at either 37 °C or 41 °C for 16 h, followed by measurement of the OD_600_. **d**, Serial dilutions of the cultures from **c** (previously incubated at either 37 °C or 41 °C) were spotted onto LB agar supplemented with chloramphenicol and incubated at 37 °C overnight before the image was taken. **e**, Serial dilutions of precultures expressing either wt CAT or CAT-K136–LOV-C450A were spotted onto LB agar supplemented with chloramphenicol and the plates were incubated overnight at the indicated temperature. **b**,**c**, Data represent the mean ± s.d. (*n* = 3 independent experiments). Statistical analysis was conducted using a two-sided Student’s *t*-test. *P < 0.05, **P < 0.01, ***P < 0.001 and ****P < 0.0001. Source Data

**Extended Data Fig. 2:**
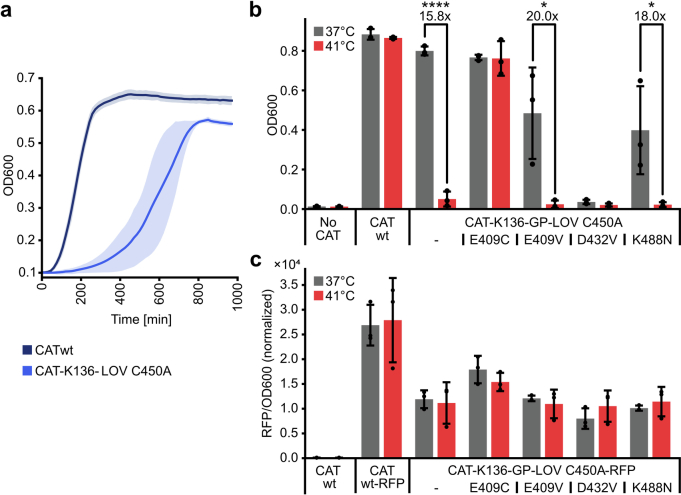
Characterization of thermosensitive CAT variants. **a**, Time course showing the growth of *E. coli* cultures expressing the indicated CAT variants at 37 °C in the presence of chloramphenicol. OD_600_ was measured every 15 minutes for 16 h using a plate reader. Lines indicate the mean of n = 3 independent replicates and shaded areas show the standard deviation. **b**, *E. coli* cultures expressing either wild-type CAT, no CAT or the indicated CAT-K136-GP-LOV mutants were grown in the presence of chloramphenicol. Replicate samples were incubated at either 37 °C or 41 °C for 16 h, followed by measurement of the OD_600_. The graph is an expansion of the dataset shown in Fig. 3c. **c**, *E. coli* cultures expressing either wild-type CAT, wild-type CAT fused to RFP or the indicated CAT-K136-GP-LOV mutants fused to RFP were grown in the absence of chloramphenicol. Replicate samples were incubated at either 37 °C or 41 °C for 16 h. RFP expression (normalized to OD) was assessed in a plate reader. **b**,**c**, Data points indicate n = 3 independent experiments and bars represent the mean. Error bars indicate the SD. Fold changes are indicated. wt, wild-type; *P < 0.05, ****P < 0.0001, two-sided Student’s t-test. Source Data

Next, to test the modularity of our thermogenetics approach, we transferred the concept to different effector proteins, starting with a chloramphenicol acetyltransferase (CAT) (Fig. 3a). Building on an existing optogenetic *As*LOV2 insertion variant at K136 of the antibiotic resistance enzyme^19^, we evaluated effects of different flexible and rigid interdomain linkers around the *As*LOV2 insertion site and assessed these variants using cell growth assays in the presence of chloramphenicol as a readout (Fig. 3b and Extended Data Fig. 2). Cultures expressing CAT–LOV hybrids reliably reached stationary phase at 37 °C, albeit at a slower growth rate than the wild-type CAT control (Extended Data Fig. 2a). Notably, for the CAT–LOV-C450A hybrid with LOV-flanking GP linkers and some *As*LOV2 mutants thereof, antibiotic resistance was effectively switched off at 41 °C resulting in cell death, as indicated by optical density at 600 nm (OD_600_) measurements (Fig. 3c and Extended Data Fig. 2b). We next verified effective bacterial killing at 41 °C by performing dilution spot assays on cultures preincubated at different temperatures (Fig. 3d). Comparable results were obtained when a preculture grown at 37 °C was spotted onto replica plates and subsequently incubated at either 37 °C or 41 °C (Fig. 3e). In the context of CAT, the K488N and E409V mutants exhibited strong thermal responses, whereas the D432V variant failed to confer antibiotic resistance at 37 °C (Extended Data Fig. 2b). As observed for AraC, CAT–LOV hybrid protein levels were substantially lower than those of wild-type CAT but were not affected by temperature, as determined by plate reader measurements of RFP-tagged CAT variants (Extended Data Fig. 2c). This finding corroborates that thermal response is driven by conformational adaptations, rather than temperature-dependent protein degradation.

To apply our method to an additional structurally unrelated protein in *E*. *coli*, we focused on a *Streptococcus* *pyogenes* Cas9 (*Spy*Cas9) CRISPR activation (CRISPRa) system, in which the transcriptional activator SoxS is recruited to a modified sgRNA scaffold by the MS2 aptamer-binding domain MCP^20,21^ (Supplementary Fig. 8a). We inserted the *As*LOV2-C450A domain into MCP at three different sites and tested these variants at 37 °C using RFP expression as a readout. A hybrid carrying the LOV insertion after N27 resulted in strong reporter expression (Supplementary Fig. 8b) and was selected for further characterization. Building on these results, we repeated the CRISPRa experiment at 37 °C and 41 °C and included the *As*LOV2-K488N mutant, which showed reduced leakiness in the context of AraC (Supplementary Fig. 8c). As expected, the LOV insertion variants were effectively inactivated at 41 °C, while the constitutively active system based on wild-type MCP was only slightly affected by the temperature increase. Consistent with our previous results, the K488N mutant reduced leakiness in the off state (41 °C), albeit at the expense of weaker activity at 37 °C (Supplementary Fig. 8c). We note that, across all systems described in this study, control samples were not affected by temperature changes, except for MCP–SoxS and AraC, where controls exhibited small but reproducible activity changes at 41 °C versus 37 °C (Fig. 1b and Supplementary Figs. 1 and 8). The readouts underlying these two cases depend on multiple protein–protein and protein–DNA interactions (DNA binding, AraC dimerization and MCP binding to MS2), which may be mildly affected by elevated temperature. This likely explains the observed, minor temperature effects in the MCP–Sox2 and AraC control samples.

Importantly, our thermogenetically regulated MCP could be easily adapted to the various applications of the MS2 aptamer system such as mRNA tagging^22^ and purification^23^ in a plug-and-play manner. Collectively, these data demonstrate the general applicability of the *As*LOV2 insertion strategy for temperature-dependent allosteric protein regulation.

### Temperature-induced peptide uncaging and temporal protein control

**Extended Data Fig. 3:**
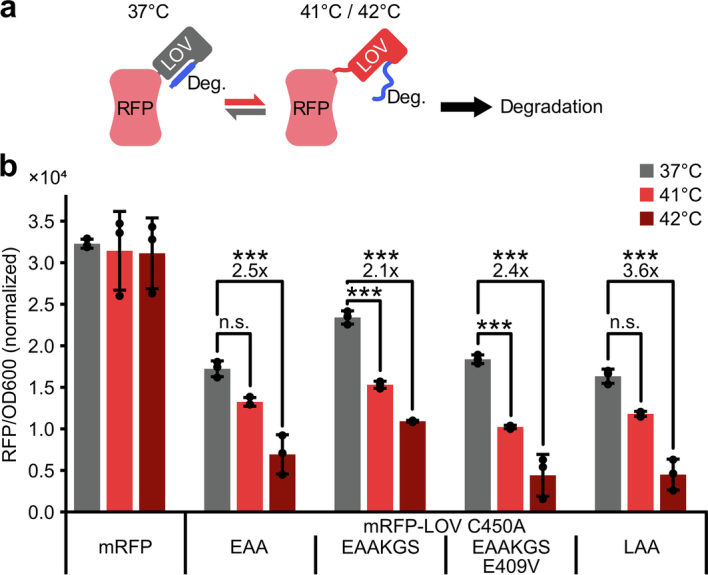
Temperature-responsive uncaging of an SsrA degron peptide in the LOV2 Jα helix. **a**, Schematic of thermo-switchable RFP degradation. **b**, *E. coli* cultures expressing either mRFP or the indicated mRFP-LOV-C450A-degron fusions were grown for 16 h at the indicated temperatures followed by measurement of RFP fluorescence and OD_600_. Letters indicate the C-terminal degron motif starting at amino acid 541 of the *As*LOV2 domain. Data points indicate n = 3 independent experiments and bars represent the mean. Error bars indicate the SD. Fold changes are indicated. n.s. P > 0.05, ***P < 0.001, one-way ANOVA with Bonferroni correction. Source Data

In addition to allosteric protein control through *As*LOV2 domain insertion, we investigated the conditional uncaging of signaling peptides in the *As*LOV2 Jɑ helix. The light-dependent exposure of functional peptides caged within the C-terminal Jα helix is a commonly used strategy in optogenetics^24–27^. In this case, the peptide is appended to or integrated into the Jα helix, resulting in peptide caging in the *As*LOV2 dark-adapted state because of the tight association of Jα with the LOV protein core. In turn, light-induced unfolding of the Jα helix exposes the peptide. To assess whether this concept could be adapted for thermoregulation, we fused RFP to several modified variants of *As*LOV2 in which the C terminus of the Jα helix was modified to resemble an SsrA degradation tag on the basis of a previously reported design^26^ (Extended Data Fig. 3a). Constitutive expression of these constructs in *E*. *coli* revealed a strong heat-dependent decrease in RFP levels at 41 °C and even more pronounced at 42 °C, while RFP expression in controls lacking the LOV-caged SsrA peptide remained unaffected by the temperature condition (Extended Data Fig. 3b).

**Extended Data Fig. 4:**
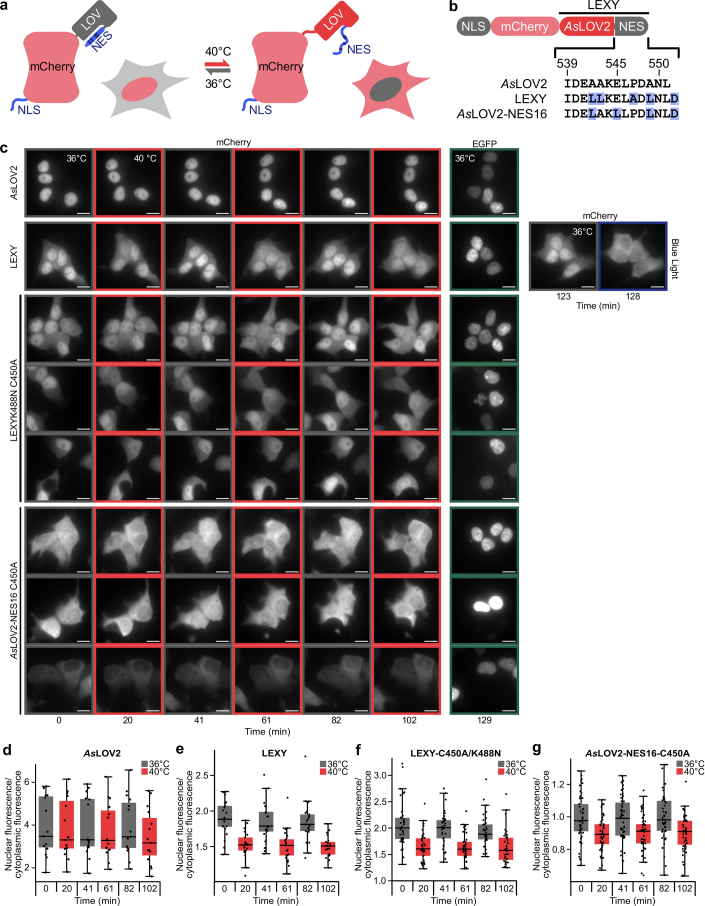
Reversible nuclear export using temperature-regulated LEXY variants. **a**, Assay schematic of LEXY system mediating temperature-regulated nuclear export. **b**, Schematic of the NLS-mCherry-LEXY and NLS-mCherry-*As*LOV2-NES16 constructs as described by Niopek et al.^2^. Mutations introduced in the Jα helix to incorporate a nuclear export signal are highlighted in blue. Residue numbers refer to full-length phototropin-1. **c**, Fluorescence microscopy images of HEK293T cells transiently transfected with plasmids encoding H2B-EGFP and mCherry fused to the indicated LEXY or *As*LOV2 variants. Post transfection, cells were incubated for 24 h at 37 °C, followed by fluorescence microscopy. Imaging was conducted during three consecutive temperature cycles, in which samples were gradually heated from 36 °C to 40 °C over a period of 20 min, followed by 20 min of cooling back to 36 °C. For canonical LEXY control images in darkness and after blue light illumination (1 s of illumination, every 60 s for 5 min) were acquired. EGFP images were acquired at the end of the experiment to visualize the nuclei. Scale bar, 13.3 µm. **d-g**, Boxplots quantifying nuclear-to-cytoplasmic fluorescence ratios of individual cells. Boxes indicate the median and interquartile range, with whiskers extending to the 1.5-fold IQR and individual data points shown. Data corresponds to n = 15 cells (**d**), n = 21 cells (**e**), n = 35 cells (**f**) and n = 47 cells (**g**). For statistical analysis of the results, see Supplementary data 1. Source Data

Complementary to the LOV–SsrA peptide degron strategy in bacteria, we implemented LEXY, a peptide-uncaging approach, for the control of protein nucleocytoplasmic trafficking in mammalian cells^25^. LEXY consists of *As*LOV2, with a nuclear export signal (NES) peptide embedded into the Jα helix, thereby enabling inducible nuclear export. When fused to a nuclear import signal (NLS)-tagged mCherry, LEXY mediates reversible nucleocytoplasmic shuttling that can be monitored in real time by fluorescence microscopy (Extended Data Fig. 4a). To assess thermal control of protein shuttling, we transiently transfected HEK293T with several LEXY variants, including the canonical LEXY, a corresponding C450A/K488N double mutant and *As*LOV2-NES16-C450A, a LEXY derivative carrying the C450A mutant and a different, functionally caged NES (Extended Data Fig. 4b). We further included a negative control construct comprising NLS–mCherry fused to wild-type *As*LOV2 instead of LEXY. After 24 h, mCherry fluorescence was imaged during repeated heating and cooling cycles consisting of 20 min of heating from 36 °C to 40 °C followed by 20 min of cooling back to 36 °C. Overall, temperature-dependent effects were substantially weaker than the light-induced nuclear export with LEXY (Extended Data Fig. 4c). Nevertheless, all three NLS–mCherry–LEXY variants consistently showed reversible heat-induced translocation to the cytoplasm, while the wild-type *As*LOV2 control remained unaffected (Extended Data Fig. 4d–g and Supplementary Data 1). The observed responses occurred on the timescale of minutes and remained reversible over three consecutive induction cycles. Together with our degron-caging experiments, these findings suggest that *As*LOV2-mediated thermoswitching is generally compatible with peptide-caging approaches but the dynamic control range is low compared to both thermocontrol through *As*LOV2 domain insertion and light-dependent peptide uncaging.

### Thermocontrol of CRISPR effectors in mammalian cells

**Fig. 4:**
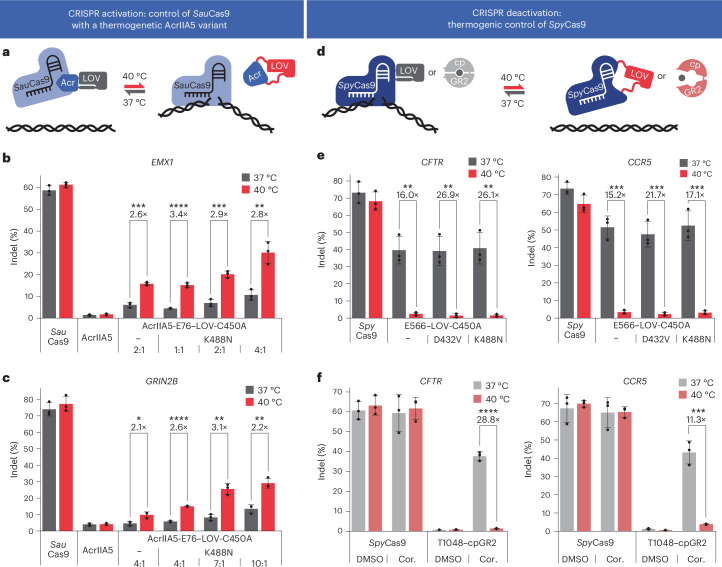
Engineering of potent thermally regulated CRISPR effectors. **a**,**d**, Assay schematics. **b**,**c**,**e**,**f**, HEK293T cells were transiently transfected with plasmids encoding *Sau*Cas9 (**b**,**c**) or the indicated *Spy*Cas9 variant (**e**,**f**), and an sgRNA targeting the indicated endogenous locus. In **b**,**c**, the respective AcrIIA5 or AcrIIA5–LOV variant or an empty stuffer plasmid was cotransfected at the indicated Cas-to-Acr vector mass ratios. Samples were incubated at 37 °C or 40 °C and editing efficiency was assessed by deep amplicon sequencing 72 h after transfection. In **b**,**c**,**e**,**f**, data are the mean ± s.d. (*n* = 3 independent experiments). Statistical analysis was conducted using a two-sided Student’s *t*-test. *P < 0.05, **P < 0.01, ***P < 0.001 and ****P < 0.0001. Source Data

Thermogenetic gene editors responding to narrow temperature changes within the human physiological range could fuel various applications in biomedical research and, eventually, precision gene therapy. Thus, we sought to transfer our concept to CRISPR–Cas9 in human cells. First, to create temperature-activated genome editors, we build upon the CASANOVA strategy for Cas9 control with light-switchable anti-CRISPR (Acr) proteins previously developed by us^28,29^. We inserted *As*LOV2-C450A or *As*LOV2-C450A/K488N after position E76 into the broad-spectrum Cas9 inhibitor AcrIIA5 (Fig. 4a) and coexpressed the resulting AcrIIA5–LOV hybrid with *Staphylococcus* *aureus* Cas9 (*Sau*Cas9) and an sgRNA targeting the endogenous *EMX1* or *GRIN2B* locus in HEK293T cells, followed by 72 h of incubation at 37 °C, 40 °C and, for *EMX1*, also 38.5 °C. We observed an up to 3.4-fold increase in indel rates between 37 °C and 40 °C at the targeted sites as determined by deep amplicon sequencing and T7 endonuclease I (T7EI) assay (Fig. 4b,c and Supplementary Fig. 9). In control samples expressing wild-type AcrIIA5 or no Acr at all, indel rates were low (<5%) or high (~60–75%), irrespective of the temperature applied (Fig. 4b,c). Consistent with our previous work on switchable Acrs, a Cas9-to-Acr vector mass ratio of 2:1 yielded the largest stimulus-dependent change in editing activity^28–30^ (Fig. 4b,c), while the flexibility of the inhibitor would also allow for case-specific optimization.

To invert this mechanism of action and demonstrate control of a second Cas9 ortholog, we directly inserted *As*LOV2 variants into *Spy*Cas9 at two allosteric sites (E566 and T1048) that we recently identified in the context of light-mediated regulation^12^ (Fig. 4d). Both Cas9–LOV hybrids mediated potent, heat-inactivated editing (Supplementary Fig. 10) with the *Spy*Cas9-E566–LOV-C450A hybrid and its *As*LOV2-D432V and *As*LOV2-K488N point mutants resulting in particularly effective thermocontrol across multiple target sites (Fig. 4e and Supplementary Fig. 11). We next assessed the effect of increased temperatures on cell viability using an MTT assay, with 72 h of incubation at 37 °C or 40 °C after transfection. As expected, cells were noticeably stressed by the transfection itself. On the contrary, the temperature condition had only minor additional effects on cell viability (Supplementary Fig. 12a), confirming that the higher temperature was, overall, well tolerated. Moreover, shortening the incubation period to 48 h further attenuated the effect of temperature on cell fitness, while still maintaining potent, temperature-dependent Cas9 activity switching (Supplementary Fig. 12b,c).

Remarkably, Cas9 thermoregulation could also be plug-and-play applied for CRISPRa simply by inserting LOV mutants at E566 into a dCas9–VPR transcriptional activator (Supplementary Fig. 13a). As a side note, an alternative CRISPRa approach using previously published ssrA-based LOV dimerization systems (iLID–ssrA, oLID–ssrA and LOV–ssrA)^31,32^ showed negligible heat-induced gene induction and only the oLID fusion responded primarily to light (Supplementary Fig. 13b).

Lastly, we hypothesized that temperature sensitivity may not be a specific feature of LOV domains only, but rather a widespread phenomenon of sensory domains. Sensory domains need to be capable of transitioning between different conformational states and may become conformationally unstable when temperature rises above their native working temperature. To investigate this hypothesis experimentally, we selected a circularly permuted glucocorticoid receptor (cpGR2) domain recently reported by us, which adopts a more compact conformation upon cortisol addition^29^. We inserted cpGR2 into *Spy*Cas9 at positions E566 and T1048 and tested the resulting hybrid effectors for temperature-dependent and ligand-dependent genome editing in HEK293T cells. In the presence of cortisol, both Cas9–cpGR2 hybrids mediated potent *CCR5* editing at 37 °C. By contrast, no editing was observed at 40 °C, irrespective of cortisol addition (Supplementary Fig. 14). The T1048 variant showed a particularly strong thermal control of genome editing across different target loci with up to 28-fold changes between the temperature conditions, whereas control samples remained unaffected (Fig. 4f). These experiments demonstrate that, like *As*LOV2, cpGR2 is inherently temperature sensitive in its cortisol-induced state.

## Discussion

We introduced the modular engineering of allosteric thermogenetic protein switches through receptor domain insertion as a powerful method for tailored protein regulation and demonstrated its applicability to diverse effectors in both bacteria and mammalian cells. Our approach leverages a commonly used photoreceptor domain and suggests that numerous other light-regulated proteins that are based on *As*LOV2 insertion could easily be adapted for temperature-dependent control. We observed multiple parallels in *As*LOV2 behavior under both stimuli, including similar mutational effects on thermal and light activation (Extended Data Fig. 1). Likewise, the same linkers between the LOV domain and the effector protein were suited for both light-driven and heat-driven control in the cases of AraC, Cas9 and AcrIIA5. However, we also noted case-specific differences, for example, the requirement for a specific linker configuration (GP instead of GS) in the CAT–LOV hybrids (Fig. 3b) and distinct effects of mutations that stabilize the native state of *As*LOV2 (Extended Data Fig. 1). These findings suggest that, although the conformational changes during light and heat induction are likely similar, subtle mechanistic differences exist that may be relevant for tailored applications.

The success of our approach raises the question of whether the temperature-dependent effects originate from intrinsic properties of the sensory domain and/or the mutual structural strain imposed by the insertion architecture. While this cannot be fully resolved on the basis of our data, the significant thermal responses observed in both tested peptide-caging configurations (LOV–SsrA and LEXY), which rely on terminal fusion of short peptides to *As*LOV2 rather than *As*LOV2 domain insertion, indicate that *As*LOV2 itself responds considerably to temperature changes (Extended Data Figs. 3 and 4). The substantially larger dynamic control ranges seen with domain insertions, however, suggest that the structural strain introduced by the insertion amplifies this thermal response (Figs. 1–4).

The fact that not only *As*LOV2 but also cpGR2 can effectively mediate thermal control may further reflect domain-specific effects but could also point toward thermosensitivity being a more widespread property among sensory domains in nature. To date, only very few proteins or domains are known to undergo changes in activity and/or conformation in response to small temperature changes close to the human physiological temperature optimum^33^. We speculate that the apparent suitability of sensory receptor domains for thermoregulation shown in our study may be explained by their specific structural and functional properties. To respond to any kind of stimulus, sensory domains must be capable of (reversibly) adopting different conformations. From a conformational energy landscape perspective, this means that the stimulus shifts the conformational equilibrium between at least two very different structural states^34^. The *As*LOV2 domain, for instance, is well known to coexist in a dark-adapted and light-adapted state but light induction strongly shifts the equilibrium toward the light-adapted conformation^35^. Importantly, the thermodynamic conformational equilibrium inherently depends on temperature. In other words, temperature changes can profoundly reshape the conformational energy landscape. This may explain why conformationally promiscuous receptor domains, especially those undergoing order–disorder transitions, may be particularly well suited for adaption as thermosensors.

In addition, the domain insertion strategy used here imposes structural strain on both the receptor domain and the effector protein. While usually not favorable for optogenetic or chemogenetic applications, the resulting destabilization of the hybrid protein is likely beneficial for the engineering of protein thermosensors, where a structural change is intended to occur at the low physiological temperatures of most biological systems^36^. This insight not only opens exciting possibilities for modularly engineering thermogenetic tools by leveraging existing optogenetic or chemogenetic switches or protein models^37^ but also underscores the importance of considering temperature effects in experimental design across biological research.

From an application perspective, the thermoregulation of CRISPR tools is of great biomedical interest but only few approaches for CRISPR–Cas effector control have been reported. While Cas9 orthologs with different thermal stabilities are known^38^, the effect of temperature changes within the physiological range on CRISPR–Cas activity tends to be negligible^39^. Similarly, the only well-characterized naturally thermosensitive Cas9 inhibitor, AcrIIA2, requires large temperature changes (that is, between 22 °C and 37 °C), which is not easily compatible with mammalian cells^40^. The only example of a directly thermocontrolled Cas9 was reported by the group of A. Möglich and is a hybrid of *Spy*Cas9 and the LOV domain from *Rhodobacter* *sphaeroides*^41^. Of note, this variant has been applied in bacteria and switches below 37 °C. Lastly, a common workaround represents the thermocontrolled expression of CRISPR–Cas using heat-shock promoters or delivery by photothermally regulated nanoparticles^6,42–44^. These systems, however, require several components and cannot be combined with, for example, cell-type-specific promoters; furthermore, heat-shock promoters are prone to crosstalk with other cellular processes.

Our system, in contrast, combines several highly advantageous features, including precise response within the small physiological temperature window, direct control of Cas9 rather than indirect transcriptional regulation, tunability of the transition temperature and the ability to provide both heat-activated and heat-inactivated control of genome editing by integrating *As*LOV2 into either Cas9 or an Acr. In addition, our strategy is modular with regard to the promoter used for expression; for example, it would be compatible with cell-type-specific promoters and should even be compatible with non-DNA-based delivery strategies (mRNA). In light of these advancements, we strongly believe that our method solves several long-standing challenges regarding thermoregulation of proteins.

## Methods

### Molecular cloning

The plasmids generated and used in this study are listed in Supplementary Table 1 and the corresponding GenBank files are provided in Supplementary Data 1. The amino acid sequences of the relevant proteins are shown in Supplementary Table 2 and the engineered variants and mutants thereof are shown in Supplementary Table 3. The constructs were generated by Golden Gate assembly, Gibson assembly or kinase, ligase and DpnI treatment. Linker sequences and type IIS restriction enzyme recognition sites were introduced as 5′ primer overhangs. Oligonucleotides were purchased from Merck and PCRs were performed using Q5 high-fidelity DNA polymerase (New England Biolabs) under standard conditions. PCR products were analyzed on agarose gels and the correct bands were excised and purified using the QIAquick gel extraction kit (Qiagen). Assembly reactions were performed using enzymes and buffers purchased from New England Biolabs and Thermo Fisher Scientific. Chemically competent *E*. *coli* Top10 cells (Thermo Fisher Scientific) were transformed with the assembled constructs and plated on agar supplemented with the appropriate antibiotic. Lastly, plasmid DNA was purified from the liquid cultures using the QIAprep Spin Miniprep or Plasmid Plus Midi kit (Qiagen). All constructs were verified by Sanger sequencing using Microsynth Seqlab. The *Renilla* luciferase expression plasmid was purchased from Promega. H2B–GFP was a gift from G. Wahl (Addgene, plasmid 11680). pX601-AAV-CMV::NLS-SaCas9-NLS-3xHA-bGHpA;U6::BsaI-sgRNA was a gift from F. Zhang (Addgene, plasmid 61591) and sgRNA1_Tet-inducible luciferase reporter and Tet-inducible mCherry reporter were gifts from M. Sato (Addgene, plasmids 64161 and 64128).

### Thermal control setup

All experiments in which samples were cultured at up to three different temperatures were performed in standard shaking incubators for bacteria or humidified incubators for mammalian cells. The incubators were operated with identical settings except for temperature, which was adjusted as indicated. Temperature settings were validated periodically using a Xintest HT-9815 thermometer and K-type thermocouples.

### AraC reporter assays

Precultures of *E*. *coli* Top10 cells transformed with constructs encoding the respective AraC variant and the mRFP reporter were inoculated from glycerol stocks and cultured overnight in 4 ml of LB medium supplemented with chloramphenicol (25 µg ml^−1^) and kanamycin (50 µg ml^−1^) at 37 °C and 220 rpm. The next day, main cultures were prepared by inoculating LB medium supplemented with chloramphenicol (25 µg ml^−1^), kanamycin (50 µg ml^−1^), 400 µM IPTG and 16 mM arabinose (unless otherwise specified) with the precultures. Cultures were grown for 16 h at 37 °C in the dark, at 37 °C under constant blue-light exposure or at 41 °C in the dark before mRFP fluorescence and OD_600_ were measured in a Tecan infinite 200 plate reader using the i-control software (Tecan, version 2.0). Except for the blue-light condition, samples were always incubated in the dark. The blue-light setup consisted of adhesive light-emitting diode strip lights (Yunbo, B50W1-B) with a wavelength of 460–465 nm attached to the metal rack tube holders in the incubator to provide continuous illumination at an intensity of 5.5–5.9 W m^−2^ as previously described^45^. For specific experiments, RFP levels of 20,000 cells were measured in a Cytoflex S flow cytometer (Beckman Coulter, Cytexpert 2.5.0.77) and analyzed using Cytoflow (version 1.3.0; https://github.com/cytoflow/cytoflow). The gating strategy is shown in Supplementary Fig. 15a,b. Initial experiments and characterization of the thermoresponsive variants were performed in 15-ml culture tubes using 4 ml of medium inoculated with 40 µl of a preculture. Dose escalation and reversibility assays were performed in 48-well plates in a 500-µl culture volume inoculated with 5 µl of preculture.

To measure the reversibility of reporter expression, precultures were diluted 1:30 in 500 µl of LB medium supplemented with all antibiotics and inducers in 48-well plates and incubated at 37 °C or 41 °C for 5 h before they were moved to the other temperature condition and incubated for another 19 h. After 3 h and 7 h of incubation, the samples were diluted 1:20 and 1:30 in fresh medium. RFP fluorescence and OD_600_ were measured at the beginning of the experiment and after 3, 7 and 24 h.

For simultaneous measurements at different temperatures, samples were incubated along a heat gradient in a thermal cycler as previously described^4^. Precultures were diluted to an OD_600_ of 0.25, inducers (400 µM IPTG and 16 mM arabinose) were added and 25-µl aliquots of the culture were divided into 12 0.2-ml PCR tubes. The tubes were placed in a thermal cycler (Eppendorf) and the gradient function was used to incubate each sample at a different temperature. After 18 h of incubation, 75 µl of LB medium was added to each sample, 90 µl of which was transferred to a 96-well microtiter plate. Fluorescence measurements were performed as described above. To infer transition temperatures, the mean reporter values for each AraC–LOV variant were min–max-normalized and fitted to the Hill equation using the Neutcurve Python package^46^.

To evaluate the spatial control of gene expression, 2.5% LB agar supplemented with chloramphenicol (25 µg ml^−1^), kanamycin (50 µg ml^−1^), 400 µM IPTG and 16 mM arabinose was prepared. Bacteria from precultures were streaked onto the agar, which was then placed in a metal tray. One half of the tray was heated from below to ~41 °C for 16 h, with the temperature of the agar monitored using an Xintest HT-9815 thermometer and a K-type thermocouple. The entire setup was kept at room temperature (24 °C) so that a steep thermal gradient was established between the heated and nonheated halves of the tray. Images were captured under blue light.

### Directed evolution experiments

To create an *As*LOV2 mutant library, the LOV domain was amplified by error-prone PCR using the GeneMorph II random mutagenesis kit (Agilent) according to the manufacturer’s protocol for medium mutation rates. The AraC expression plasmid was opened at position S170 by around-the-horn PCR using Q5 HotStart high-fidelity DNA polymerase (New England Biolabs). The primers carried type IIS restriction enzyme recognition sites as overhangs enabling the efficient assembly of the AraC-S170–LOV fusion sequence through Golden Gate cloning. After Golden Gate assembly, electrocompetent *E*. *coli* Top10 cells carrying a pBAD-driven mRFP reporter were transformed with the plasmid library using a Gene Pulser Xcell (Biorad) at 1,800 V and 200 Ω. The transformed cells were allowed to recover for 1 h in 1 ml of SOC medium without antibiotics while incubating at 37 °C and 800 rpm in a thermoshaker (Eppendorf). Next, a 10-ml culture of LB medium supplemented with chloramphenicol (25 µg ml^−1^) and kanamycin (50 µg ml^−1^) was inoculated with the whole 1 ml of culture and grown overnight. In parallel, serial dilutions of the transformants were plated on agar and used to determine a transformation efficiency of 5.2 million colony-forming units. Sanger sequencing of randomly picked colonies revealed an amino acid substitution rate of ~1.3 per clone. Considering a theoretical library complexity of 2,679 possible single amino acid mutants and 7.2 million double mutants, all single mutants and many of the possible double mutants were covered. Lastly, glycerol stocks of the library were prepared in aliquots and stored at −80 °C until further use.

To select the library for thermoresponsive candidates, a preculture was grown from the glycerol stocks overnight at 37 °C and 220 rpm. The next day, the cultures were diluted 1:1,000,000 and 400 µl were plated onto 20 large LB agar petri dishes (20-cm diameter) supplemented with chloramphenicol (25 µg ml^−1^), kanamycin (50 µg ml^−1^), 400 µM IPTG and 16 mM arabinose. Plates were incubated overnight at 40 °C and photographed under blue light. The plates were then incubated at 37 °C for an additional 2 h and imaged again. Next, pictures were analyzed computationally the ‘colony counter’ package (https://github.com/morris-lab/Colony-counter). Colonies that were nonfluorescent after the initial incubation at 40 °C but showed bright fluorescence after the 37 °C incubation step were considered promising candidates. These clones were picked and their performance was tested using the quantitative reporter assay described above. Mutations in the verified lead candidates were determined by Sanger sequencing.

### Western blot

Cultures were prepared as described for AraC reporter assays. Then, 1 ml of culture was pelleted at 20,000*g* for 2 min and resuspended in 300 µl of water. The samples were then mixed with 100 µl of 4× Laemmli sample buffer (BioRad) supplied with 10% β-mercaptoethanol and incubated at 95 °C for 10 min to lyse the cells, followed by centrifugation at 20,000*g* for 2 min. Next, 25 µl of the sample was loaded onto a NuPAGE 12%, Bis–Tris gel (Thermo Fisher Scientific) and PAGE was performed at 200 V for 35 min in MES buffer. The proteins were transferred onto a PVDF membrane overnight at 100 V and 10 °C. The membrane was blocked for 1 h at room temperature with 4% milk powder in Tris-buffered saline with Tween-20 (TBS-T), followed by an overnight incubation with a mouse anti-His IgG primary antibody (Proteintech, 66005-1-Ig; 1:50,000) at 10 °C. The next day, the membrane was washed three times for 10 min with TBS-T and was subsequently incubated with a goat anti-mouse IgG secondary antibody (Jackson Immuno Research, 115-035-068; 1:10,000) for 1 h at room temperature, followed by three additional TBS-T washes. The western blot was developed using SuperSignal West Pico PLUS (Thermo Fisher Scientific) for 5 min before images were acquired (LAS 3000 Imaging System, Fuji).

### Antibiotic resistance assays

Precultures of *E*. *coli* Top10 cells transformed with the respective CAT-encoding construct were inoculated from glycerol stocks and grown overnight in 4 ml of LB medium supplemented with ampicillin (100 µg ml^−1^) at 37 °C and 220 rpm. The next day, main cultures were prepared by adding 5 µl of precultures to 48-well plates containing 500 µl of LB medium supplemented with 100 µg ml^−1^ ampicillin and 25 µg ml^−1^ chloramphenicol in technical duplicates. Two plates were prepared, one incubated at 37 °C and 220 rpm and the other incubated at 41 °C and 220 rpm. After 16 h of incubation, the OD_600_ was measured in a Tecan infinite 200 plate reader. The values of an LB-only control were subtracted from each sample. For quantitative assessment of cell survival, serial dilutions of main cultures grown at different temperatures were subsequently spotted onto LB agar supplemented with 100 µg ml^−1^ ampicillin and 25 µg ml^−1^ chloramphenicol and grown overnight at 37 °C before images were taken. Thermally induced killing of bacteria growing on agar was performed in a similar manner. In this case, serial dilutions of a preculture were spotted onto two separate replicate LB agar plates supplemented with 100 µg ml^−1^ ampicillin and 25 µg ml^−1^ chloramphenicol, one incubated overnight at 37 °C and the other incubated at 41 °C.

To measure growth curves, cultures were prepared identically but incubated in the plate reader at 37 °C for 16 h, while shaking at a 4-mm radius. The OD_600_ was measured every 15 min. To measure RFP fluorescence, precultures of *E*. *coli* Top10 cells transformed with constructs encoding the respective CAT–RFP variant were inoculated from glycerol stocks. These cultures were then grown overnight in 4 ml of LB medium supplemented with ampicillin (100 µg ml^−1^) at 37 °C and 220 rpm. The next day, main cultures were prepared by inoculating 4 ml of LB medium supplemented with ampicillin (100 µg ml^−1^) with 40 µl of the precultures. After 16 h of incubation at either 37 °C or 41 °C, mRFP fluorescence and OD_600_ were measured in a Tecan Infinite 200 plate reader. All incubation steps were performed in the dark.

### Inducible protein degradation

*E*. *coli* Top10 cells were transformed with plasmids encoding different mRFP–LOV fusion reporter proteins. Precultures were inoculated from glycerol stocks and grown overnight at 37 °C and 220 rpm in 4 ml of LB medium supplemented with (50 µg ml^−1^) kanamycin. The next day, three identical main cultures were prepared by inoculating 4 µl of the respective precultures into 4 ml of LB medium supplemented with the same antibiotic and 1 mM arabinose, followed by incubation at 220 rpm and 37, 41 or 42 °C. After 16 h of incubation, mRFP fluorescence and OD_600_ were measured in a Tecan infinite 200 plate reader. All incubation steps were performed in the dark.

### SoxS-mediated CRISPRa in *E*. *coli*

*E*. *coli* Top10 cells were cotransformed with plasmids encoding an mRFP reporter and d*Spy*Cas9, an sgRNA with an MS2 stem loop integrated into its scaffold and an MCP–SoxS fusion protein or variants thereof. Precultures were inoculated from glycerol stocks and grown overnight at 37 °C and 220 rpm in LB medium supplemented with 100 µg ml^−1^ ampicillin and 25 µg ml^−1^ chloramphenicol. The next day, two identical main cultures were prepared by inoculating 4 ml of LB medium supplemented with the same antibiotics with 100 µl of the respective precultures, followed by incubation at 220 rpm and 37 or 41 °C. All incubation steps were performed in the dark. After 16 h of incubation, mRFP fluorescence and OD_600_ were measured in a Tecan infinite 200 plate reader. The values of an LB-only control were subtracted from each sample.

### Human cell culture

HEK293T cells were maintained in 1× DMEM without phenol red (Thermo Fisher Scientific) supplemented with 10% (v/v) FBS (Thermo Fisher Scientific), 2 mM L-glutamine, 100 U per ml penicillin and 100 μg ml^−1^ streptomycin (all from Thermo Fisher Scientific). Cells were cultured at 37 °C with 5% CO_2_ in a humidified incubator and passaged at a confluency of 70–80%. Authentication of the cell line was performed before use and routine testing for *Mycoplasma* contamination was conducted. Cell growth at 40 °C was visually inspected with a Keyence BZ-9000 microscope using the Keyence BZ-II Viewer (Keyence, version 1.01) and BZ-II Analyzer software (Keyence, version 1.01) (Supplementary Fig. 16). Cells were always incubated in the dark, only being exposed to ambient light during transient transfection and immediately before lysis. HEK293T cells were provided by E. Wiedtke (Heidelberg University clinics and BioQuant). The cell line was authenticated by single-nucleotide polymorphism profiling (Multiplexion) in March 2025.

### Transient transfection

HEK293T cells were seeded at a density of 12,500 cells per well in 100 µl of medium into a 96-well plate. After 24 h, cells were transfected with a total of 150 ng of DNA using 0.5 µl of Lipofectamine 2000 (Thermo Fisher Scientific) per well according to the manufacturer’s instructions. For genome editing using *Spy*Cas9, cells were cotransfected with 75 ng of a construct expressing either wild-type *Spy*Cas9 or a *Spy*Cas9–LOV or *Spy*Cas9–cpGR2 hybrid and 75 ng of the corresponding sgRNA expression vector. For AcrIIA5-dependent genome editing with *Sau*Cas9, cells were cotransfected with 150 ng of (1) an all-in-one construct encoding *Sau*Cas9 and an *EMX1*-targeting or *GRIN2B*-targeting sgRNA and (2) AcrIIA5 wild type or the AcrIIA5-E76–LOV2 mutant at the vector mass ratios indicated in the figures. Cas9 without sgRNA was transfected as a negative control. The sgRNA sequences used in this study are listed in Supplementary Table 4. After transfection, all samples were either incubated for 72 h at 37 °C or 40 °C with 5% CO_2_ in a humidified incubator. For the chemical induction, 1 µl of 20 µM cortisol was added per well after 2 h, where 200 µM of cortisol dissolved in DMSO was prediluted tenfold in DMEM.

### Imaging of nuclear export using LEXY

HEK293T cells were seeded into four-compartment 35-mm dishes at a density of 75,000 cells per chamber in 500 µl of medium. After 24 h, each chamber was transfected with 250 ng of DNA using 0.75 µl of Lipofectamine 2000 (Thermo Fisher Scientific). Cells were cotransfected with 20 ng of a construct encoding H2B–EGFP, 10 ng of a construct encoding mCherry fused to *As*LOV2 or a LEXY variant, as well as 220 ng of a stuffer plasmid. Then, 24 h after transfection, 200 µl of medium was added to each chamber.

Fluorescence imaging was performed at the Nikon Imaging Center at Heidelberg University with a Nikon total internal reflection fluorescence microscope, equipped with a Nikon S Fluor ×40 objective, an Andor Neo 5.5 scientific complementary metal–oxide–semiconductor camera and an on-stage incubation chamber (TokaiHit). To prevent exposure to ambient light, cells were kept in the dark and focused on the mCherry channel. For time-lapse microscopy, an incubation chamber was gradually heated from 36 °C to 40 °C over a period of 20 min, followed by 20 min of cooling back to 36 °C. CO_2_ levels were constantly kept at 5%. mCherry images were taken every 2 min (excitation wavelength: 560 nm, emission wavelength: 620 nm, 27% laser intensity) with a 400-ms exposure time. Subsequently, LEXY samples were exposed to 1 s of blue-light irradiation every minute for 5 min (excitation wavelength: 475 nm, emission wavelength: 525 nm, 100% laser intensity) to measure blue-light response. EGFP imaging was performed in the same FITC channel with 17% laser intensity and 400-ms exposure time.

To quantify the nuclear and cytoplasmic mCherry fluorescence, images were first bleach-corrected using the simple ratio method in Fiji 2.16.0. Background fluorescence was subtracted using the mean intensity of three regions of interest (ROIs) with a 100-pixel diameter. Subsequently, 30-pixel diameter ROIs were manually assigned in the nucleus and cytoplasm to calculate fluorescence intensities. Statistical tests for all groups are provided in Supplementary Data 1.

### Genome editing of endogenous loci

Editing efficiency at endogenous loci was assessed by deep amplicon sequencing or T7EI assay, as indicated. In both cases, samples were processed 72 h after transfection. Media were aspirated and cells were lysed in DirectPCR lysis reagent (PeqLab) supplemented with 200 µg ml^−1^ proteinase K (Roche Diagnostics). Lysis was performed in a shaking incubator at 120 rpm and 55 °C for at least 6 h, followed by heat inactivation of proteinase K at 85 °C for 45 min. Targeted genomic loci were PCR-amplified using Q5 high-fidelity 2× master mix (New England Biolabs) with the primers listed in Supplementary Table 5. Dual-barcoded versions of these primers were used for next-generation sequencing. Correct amplicon length and purity were determined by running samples on a 1% 0.5× Tris–acetate–EDTA agarose gel. Barcoded samples were pooled in equimolar ratios and Illumina sequencing was performed by GENEWIZ using the Amplicon-EZ service. Indel frequencies were assessed using CRISPresso 2.0 (version 2.3.1) as previously described (https://github.com/pinellolab/CRISPResso2, https://github.com/mjendrusch/acr-dms)^47,48^. For T7EI assays, 5 µl of PCR amplicons were annealed in 20 µl of 1× NEB buffer 2 by heating the samples to 95 °C for 5 min, followed by gradual cooling to 25 °C. The annealed samples were incubated with 0.5 µl of T7EI at 37 °C for 15 min. Gene editing efficiency was quantified by running samples on a 2% 1× Tris–borate–EDTA agarose gel and analyzing band intensities using Fiji 2.16.0. The editing efficiency was calculated as 100 × (1 − √(1 − cleaved fraction)), where the cleaved fraction is the sum of the intensities of the cleaved bands divided by the total band intensities. Uncropped gel images are provided in Supplementary Fig. 17.

### d*Spy*Cas9–VPR-mediated CRISPRa in mammalian cells

HEK293T cells were seeded at a density of 75,000 cells per well in 600 µl of medium into a 24-well plate. After 24 h, cells were transfected with a total of 600 ng of DNA using 1.8 µl of Lipofectamine 3000 (Thermo Fisher Scientific) according to the manufacturer’s instructions. Cells were cotransfected with (1) 240 ng of a construct encoding d*Spy*Cas–VPR wild type or d*Spy*Cas–LOV–VPR hybrid; (2) 120 ng of a TetO-targeting sgRNA expression vector; (3) 120 ng of a 13×TetO–mCherry–MODC reporter plasmid coencoding a constitutively expressed EGFP–MODC as transfection control; and (iv) 120 ng of stuffer plasmid. For the iLID-based transcriptional activation assay, the cells were cotransfected with (1) 360 ng of a plasmid encoding d*Spy*Cas–VPR or 180 ng of plasmids encoding d*Spy*Cas–LOV2ssrA/oLID/iLID and sspB–VPR; (2) 120 ng of a TetO-targeting sgRNA expression vector; and (3) 120 ng of a 13×TetO–mCherry–MODC reporter construct. Cells were incubated at either 37 °C or 40 °C for 48 h. For the iLID experiments, a third condition was included, in which the cells were incubated at 37 °C under blue-light illumination. The light setup was controlled by a Raspberry Pi running a custom Python script that applied an illumination duty cycle of 5 s of light followed by 10 s of darkness. The light intensity was set to 30 μmol m^−2^ s^−1^. For the flow cytometry assay, the cells were washed with 1× PBS, trypsinized and resuspended in 1× DMEM without phenol red supplemented with 10% (v/v) FBS, 2 mM L-glutamine, 100 U per ml penicillin and 100 μg ml^−1^ streptomycin (all from Thermo Fisher Scientific). The cells were transferred to 2-ml tubes and kept on ice from this stage onward. The samples were washed with 800 µl of PBS, resuspended in 250 µl of PBS and passed through a 0.45-µm cell strainer. Flow cytometry was carried out on a Cytoflex S (Beckman Coulter, using Cytexpert 2.5.0.77.). Cells were identified using the forward scatter and side scatter channels as indicated in Supplementary Fig. 15c,d. For each sample, 20,000 cells were recorded. The mCherry fluorescence signal was measured with a yellow laser (excitation: 561 nm, emission: 610 nm). The collected data were subsequently processed and analyzed using the Cytoflow software package (version 1.3.0; https://github.com/cytoflow/cytoflow).

### Cell viability assay

HEK293T cells were seeded and transfected in 96-well plates, as described above. Then, 48 h or 72 h after transfection, Triton X-100 was added to the untreated wells at concentrations ranging from 1% to 0.001% as a positive control for cell death.

The cell viability assay was performed using the MTT cell proliferation assay kit (Cayman Chemicals) following the manufacturer’s instructions. Absorbance was measured at 570 nm using an Infinite M Plex microplate reader (Tecan).

### Reporting summary

Further information on research design is available in the Nature Portfolio Reporting Summary linked to this article.

## Online content

Any methods, additional references, Nature Portfolio reporting summaries, source data, extended data, supplementary information, acknowledgements, peer review information; details of author contributions and competing interests; and statements of data and code availability are available at 10.1038/s41589-026-02151-y.

## Supplementary information

Supplementary Information Supplementary Figs. 1–17, Supplementary Tables 1–5 and Supplementary References.

Reporting Summary

Supplementary Data 1 Raw data supplementary figures, GenBank files, structures, T7 gels and western blot images.

## Source data

Source Data Figs. 1–4 and Extended Data Figs. 1–4 Raw and statistical data.

## Data Availability

Additional information, including relevant amino acid sequences, targeted genomic loci and PCR primers for indel quantification are provided in the Supplementary Information. GenBank files of the DNA constructs are provided in Supplementary Data 1. Important constructs will be shared through Addgene (250776–250780). Source data are provided with this paper.
