# Supplementary material for: Modular engineering of thermoresponsive allosteric proteins
Source: Nat Chem Biol. 2026 Feb 12;22(5):751–8. doi: 10.1038/s41589-026-02151-y (PMC13128441; doi:10.1038/s41589-026-02151-y)
Supplement: Supplementary file 1 — Supplementary Figs. 1–17, Supplementary Tables 1–5 and Supplementary References. [file 41589_2026_2151_MOESM1_ESM.pdf]

nature chemical biology

Supplementary information

https://doi.org/10.1038/s41589-026-02151-y

Modular engineering of thermoresponsive allosteric proteins

In the format provided by the authors and unedited

Content:

Supplementary Figure 1-17

Supplementary Table 1-5

Supplementary References

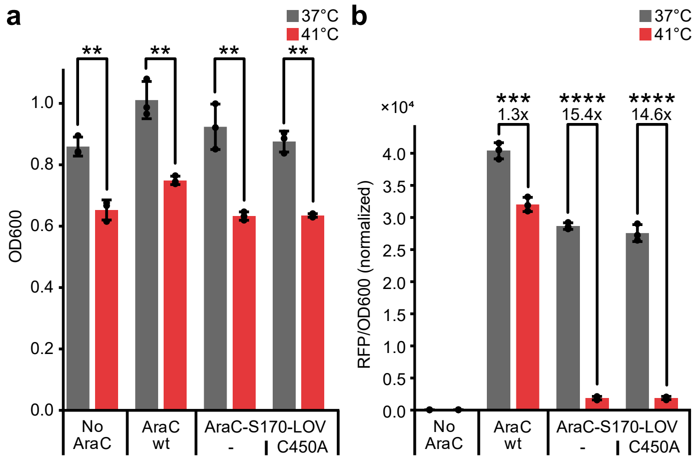

Supplementary Figure 1 | Insertion of *As*LOV2 into AraC enables thermo-switchable gene expression in *E. coli*. a, b, *E. coli* carrying a pBAD-mRFP reporter and expressing the indicated AraC variant or a dummy control protein of similar size were incubated at 37°C or 41°C for 16 h. Culture density (OD600) (**a**) and RFP expression (normalized to OD) (**b**) were assessed in a plate reader. Data points indicate n=3 independent experiments and bars represent the mean. Error bars indicate the standard deviation (SD). Fold changes are indicated. wt, wild-type; **P < 0.01, ***P < 0.001, ****P < 0.0001, two-sided Student’s t-test.

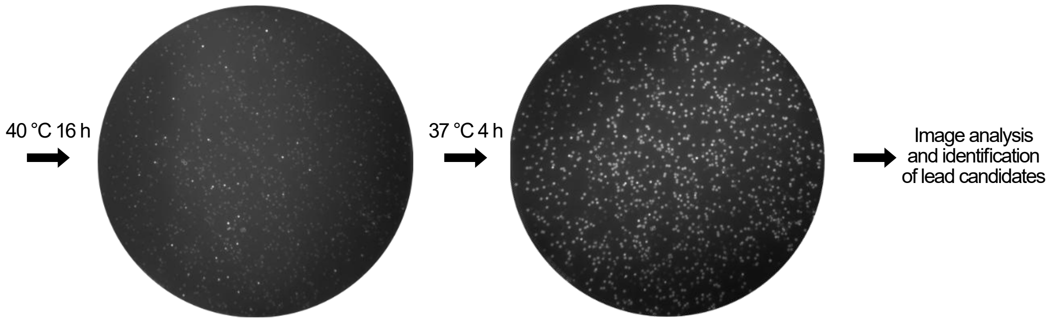

Supplementary Figure 2 | Selection of improved, thermo-switchable AraC-LOV2 hybrid mutants via screening on agar plates. Workflow of the AraC-S170-LOV library screening and corresponding agar plate images. Images of the same agar plate were acquired under blue light illumination, first after an initial overnight incubation at 41°C and then after an additional 4 hours at 37°C. Colonies showing no/low fluorescence at 40°C and high fluorescence at 37°C indicate variants with potent photoswitching and were therefore selected for downstream analysis.

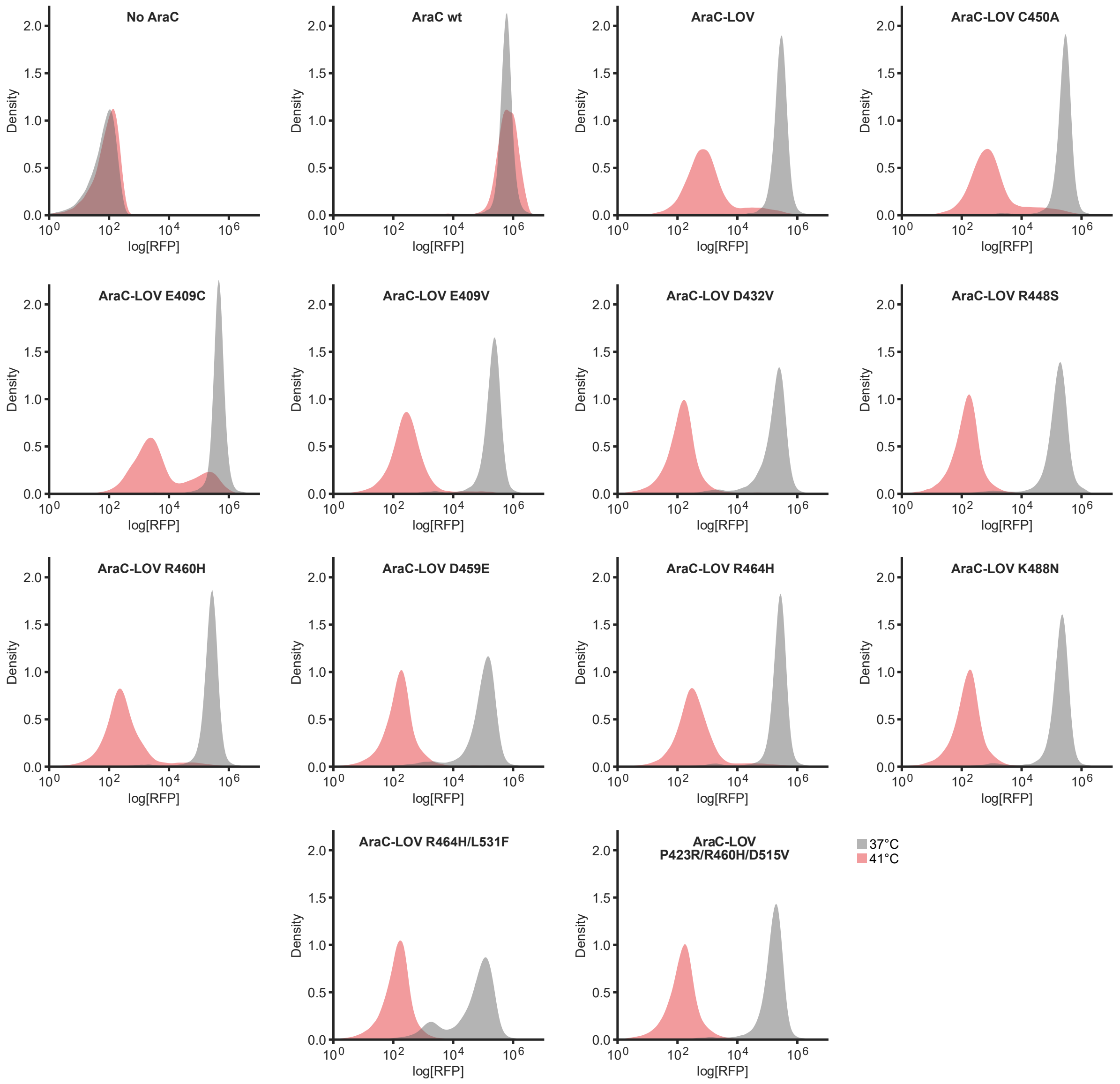

Supplementary Figure 3 | Directed evolution yields several potent thermosensitive LOV variants. *E. coli* carrying an AraC reporter and expressing AraC, AraC-LOV, the indicated AraC-LOV-C450A variant or a dummy protein as control were grown at 37°C or 41°C for 16 h, followed by flow cytometry analysis. Histograms show the distribution of fluorescence within the respective sample population for n=3 pooled independent replicates. wt, wild-type.

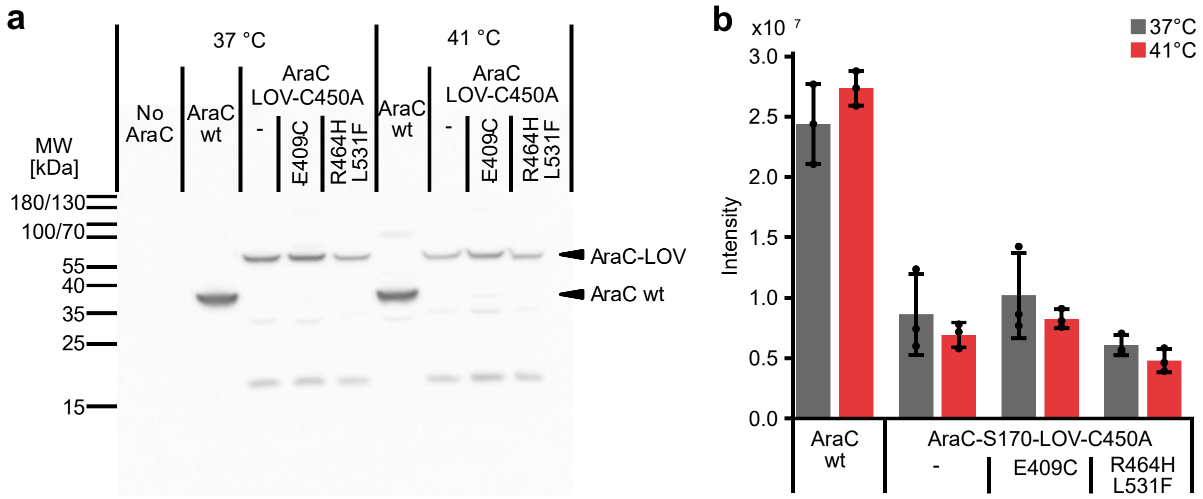

Supplementary Figure 4 | AraC-LOV protein levels remain unaffected by different temperature conditions. a,b, Western blots of selected AraC hybrids. *E. coli* expressing a pBAD-mRFP reporter and the indicated His-tagged AraC variant or a dummy control protein of similar size were incubated at 37°C or 41°C for 16 h. Subsequently, protein levels were assessed by Western blot. **a**, Representative Western blot image. Bands corresponding to AraC wt and AraC-LOV fusions are indicated. **b**, Quantification of AraC and AraC-LOV band intensities. Data represent the mean +/- SD, n = 3 independent experiments. wt, wild-type.

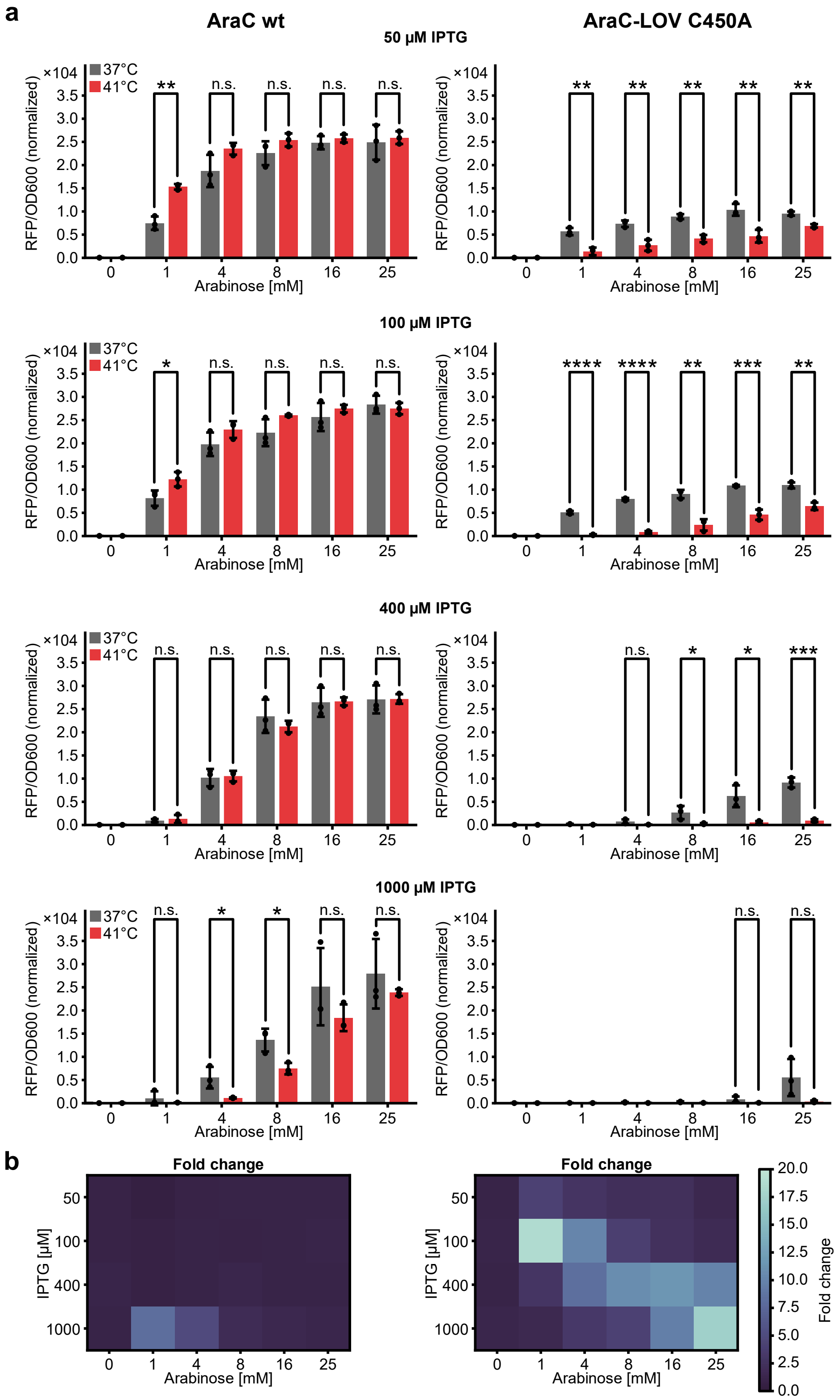

Supplementary Figure 5 | The thermal response of AraC-LOV can be tuned by varying inducer concentrations. a, Raw data corresponding to the dose escalation screen in Fig. 2a. *E. coli* carrying a pBAD-mRFP reporter and AraC wt or AraC-LOV-C450A were incubated for 16 h at 37°C or 41°C in the presence of inducers at the indicated concentration. Expression of the AraC variants is IPTG-inducible, while AraC

activity is arabinose-dependent. RFP fluorescence and OD600 were measured in a plate reader. Data represent the mean +/- SD, n = 3 independent experiments. **b**, Fold changes between the 37°C and 41°C conditions of the data presented in **a** are shown. wt, wild-type; n.s. $P > 0.05$, *$P < 0.05$, **$P < 0.01$, ***$P < 0.001$, ****$P < 0.0001$, two-sided Student's t-test.

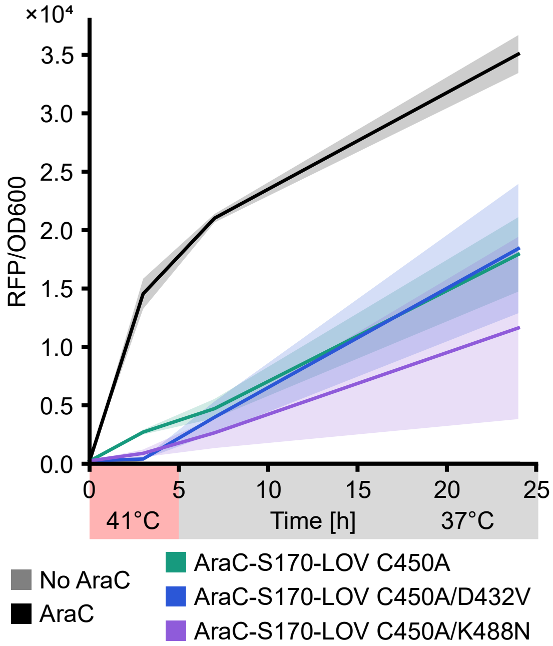

Supplementary Figure 6 | Temporal control of gene expression. *E. coli* containing a pBAD-mRFP reporter and expressing the indicated AraC variant or a dummy control protein were incubated at 41°C for 5 h followed by another 19 h of incubation at 37°C. Culture density (OD600) and RFP expression (normalized to OD) were periodically assessed in a plate reader. Data corresponds to the mean of n=3 independent experiments. The SD is indicated.

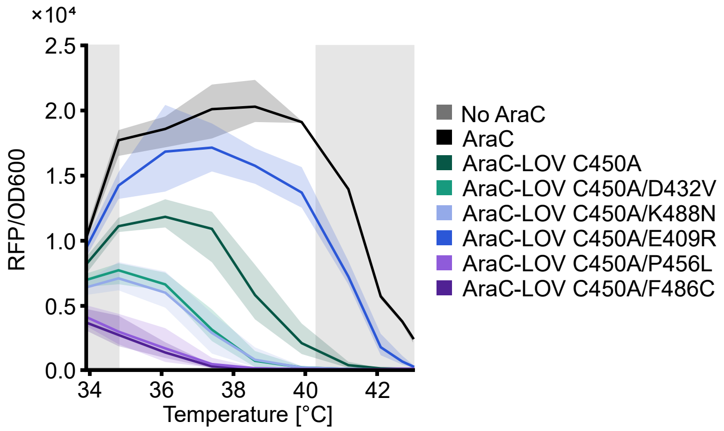

Supplementary Figure 7 | Point mutations tune the transition temperature and amplitude of thermogenetic AraC. *E. coli* encoding the pBAD-mRFP reporter and the indicated AraC variant or a control without AraC were incubated for 16 h at different temperatures between 34°C and 43°C followed by measurement of RFP fluorescence and OD600 in a plate reader. Lines represent the mean of n=3 independent biological replicates. Shaded areas indicate the SD. Regions marked in gray indicate reduced accuracy of the assay due to significant decrease in AraC wild-type reporter levels at these *E. coli* growth conditions. Data represents the raw values corresponding to Fig. 2e.

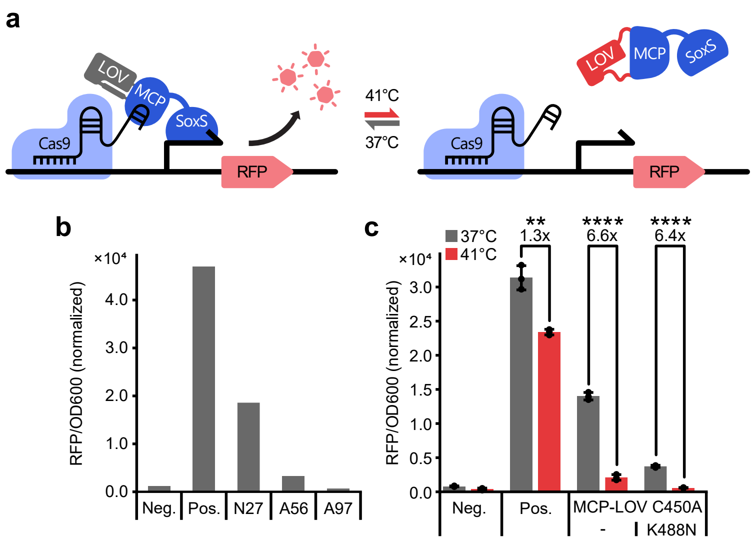

Supplementary Figure 8 | Screening of LOV insertion sites in MCP. a, Schematics of MCP-SoxS mediated CRISPRa**. b**, *E. coli* were transformed with plasmids encoding a CRISPRa circuit, i.e. (i) dCas9, (ii) a promoter-targeting or non-targeting sgRNA harboring an MS2 stem loop in its scaffold sequence, (iii) the MCP-SoxS transactivator with an *As*LOV2-C450A insertion after the indicated MCP residue, and (iv) an mRFP reporter. Cultures were incubated at 37°C for 16 h followed by measurement of mRFP fluorescence and OD600. Bars represent a single experiment. **c**, *E. coli* encoding the same circuit as in b, including the MCP-N27-LOV insertion variant or a K488N point mutant thereof were incubated at 37°C or 41°C for 16 h followed by measurement of mRFP fluorescence and the OD600. Data points indicate n=3 independent replicates and bars represent the mean. Error bars indicate the SD. Fold changes are indicated. Neg., non-targeting negative control; Pos., positive control expressing an mRFP-targeting sgRNA and MCP-SoxS without LOV insertion. **$P < 0.01$, ****$P < 0.0001$, two-sided Student's t-test.

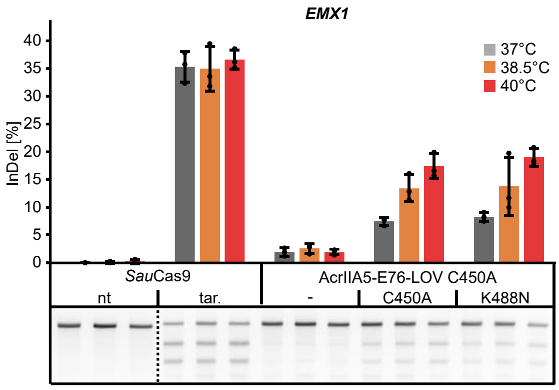

Supplementary Figure 9 | Control of *EMX1* locus editing across different temperatures. HEK293T cells were transiently transfected with plasmids encoding (i) wild-type *Sau*Cas9 with a sgRNA targeting *EMX1* and (ii) the respective AcrIIA5(-LOV) variant in a 2:1 Cas:Acr vector mass ratio. Replicate samples were incubated at 37°C, 38.5°C or 40°C, respectively, and editing efficiency was assessed 72 h post-transfection via T7EI assay. Data represent the mean +/- SD, n = 3 independent experiments. A representative agarose gel image is shown below the graph. nt, non-targeting; tar., targeting.

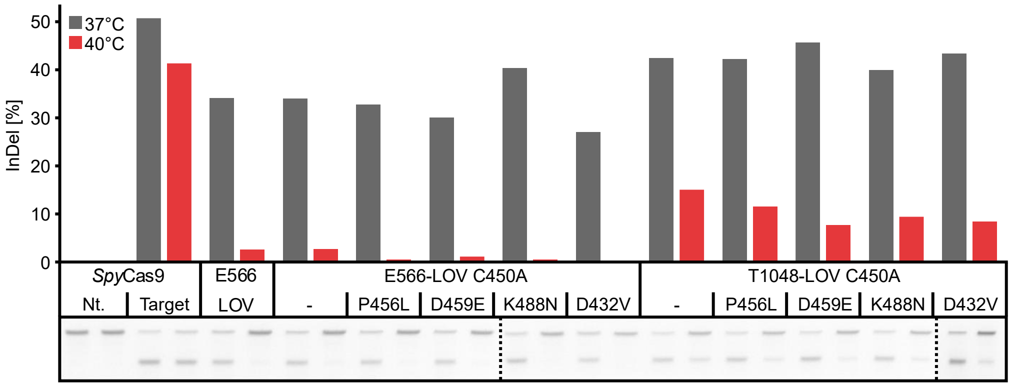

Supplementary Figure 10 | Screening for thermosensitive *Spy*Cas9 variants. HEK293T cells were transfected with plasmids encoding (i) an sgRNA targeting the endogenous *CCR5* locus or a non-targeting control and (ii) the indicated Cas9-LOV fusion variant or a wild-type Cas9 control and incubated at 37°C or 40°C. 72 h post-transfection, editing efficiencies were assessed in a T7EI assay. Bars indicate InDel frequencies from a single experiment, calculated on the basis of the corresponding gel images (bottom). Nt., non-targeting.

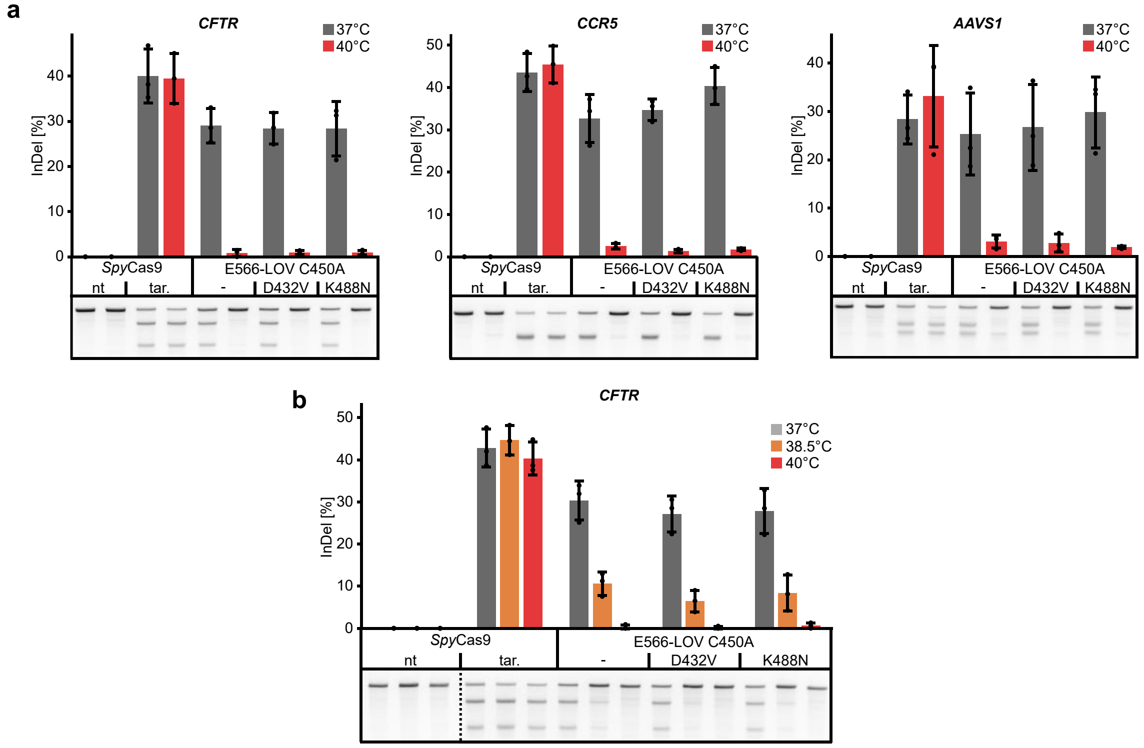

Supplementary Figure 11 | Effective thermal control of genome editing using *Spy*Cas9-LOV hybrids. **a**, Indel quantification of the samples corresponding to Fig. 4h by T7EI assay. Additionally, data for a third genomic locus, *AAVS1*, is shown for the Cas-LOV constructs. **b**, HEK293T cells were transiently transfected with plasmids encoding (i) wild-type *Spy*Cas9 or the indicated *Spy*Cas9 variants with an *As*LOV2 insertion after E566, as well as a (ii) sgRNA targeting *CFTR*. Replicate samples were incubated at 37°C, 38.5°C or 40°C as indicated, and editing efficiency was assessed 72 h post-transfection via T7EI assay. **a,b**, Data represent the mean +/- SD, n = 3 independent experiments. A representative agarose gel image is shown below each graph. nt, non-targeting; tar., targeting.

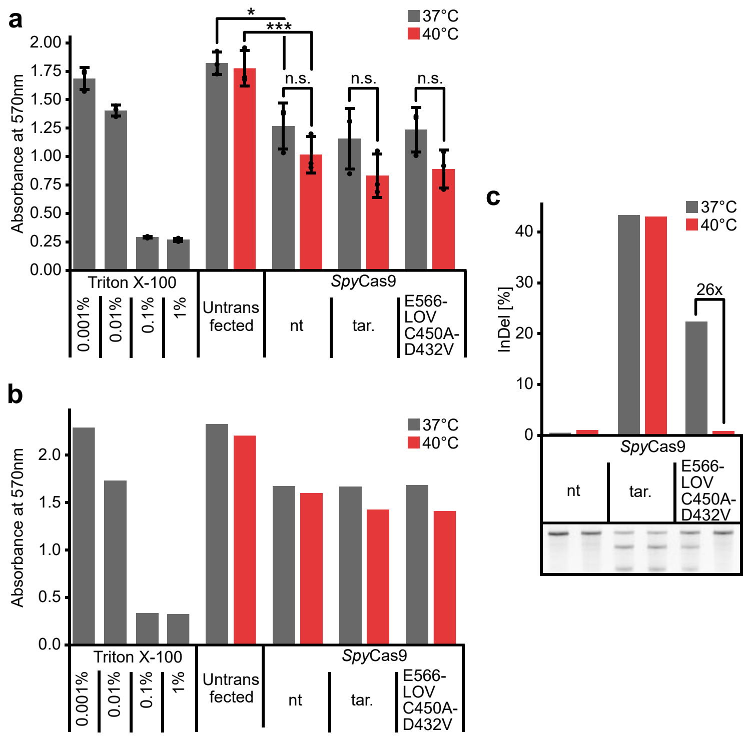

Supplementary Figure 12 | Influence of transient transfection and temperature conditions on cell viability. a,b, HEK293T cells were transiently transfected with *Spy*Cas9 or the *Spy*Cas9-E566-C450A/D432V variant and a sgRNA targeting the endogenous *CFTR* locus or a non-targeting sgRNA as negative control. Samples were incubated at 37°C or 40°C for 72 h (**a**) or 48 h (**b,c**) before cell viability was assessed by an MTT assay. As an assay control, cells were treated with different concentrations of toxic Triton X-100. Data represents the mean +/- SD of n=3 independent experiments (**a**) or the mean of n=3 technical replicates (**b**). **c**, HEK293T cells were transiently transfected with *Spy*Cas9 or the *Spy*Cas9-E566-C450A/D432V variant and a sgRNA targeting the endogenous *CFTR* locus or a non-targeting sgRNA as negative control. Samples were incubated at 37°C or 40°C for 48 h before genome editing efficiency was assessed using a T7EI assay. Bars represent a single experiment. The corresponding agarose gel image is shown. Fold changes are indicated. **a-c**, nt, non-targeting; tar., targeting; a.u., arbitrary units. n.s. P > 0.05, *P < 0.05, ***P < 0.001, one-way ANOVA with Bonferroni correction.

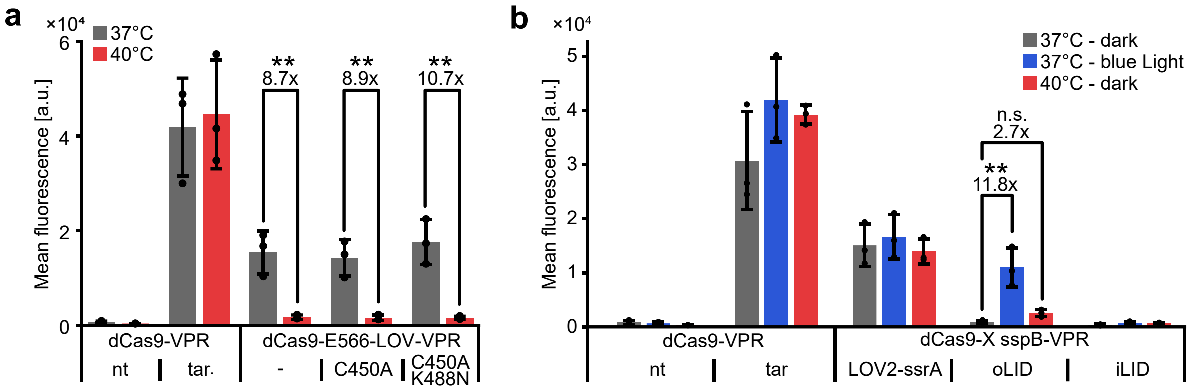

Supplementary Figure 13 | Temperature-regulated transcriptional activation. a,b, HEK293T cells were transiently transfected with plasmids encoding (i) d*Spy*Cas9-VPR or the corresponding *As*LOV2 insertion variants (**a**), or d*Spy*Cas9 fused to the modified LID version and sspB-VPR (**b**), (ii) an mCherry reporter driven from a minimal promoter preceded by 13x TetO repeats, and (iii) a TetO-targeting sgRNA. Samples were incubated at the indicated temperature or light conditions for 48 h before mCherry fluorescence was assessed by flow cytometry. Data represent the mean +/- SD, n = 3 independent experiments. Fold changes are indicated. nt, non-targeting; tar., targeting; a.u., arbitrary units; **a**, **P < 0.01, two-sided Student's t-test. **b**, n.s.P > 0.05, **P < 0.01, one-way ANOVA with Bonferroni correction.

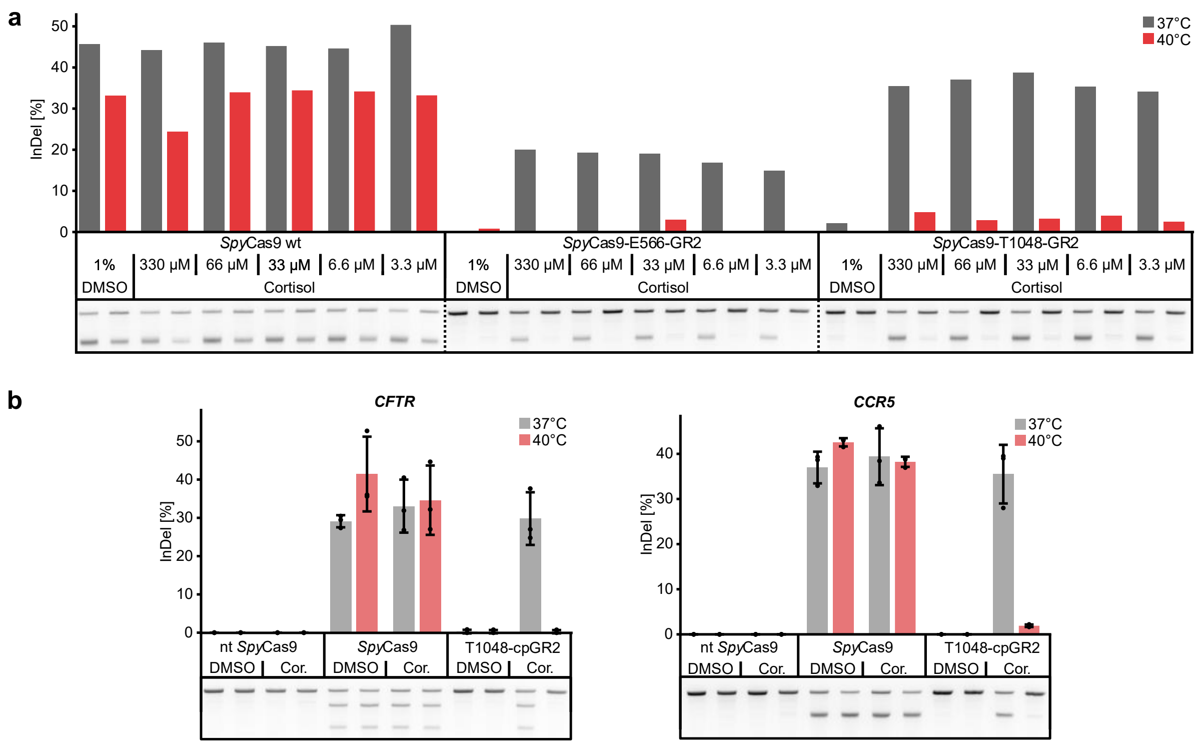

Supplementary Figure 14 | Temperature-responsive genome editing with *Spy*Cas9-GR2 hybrids. a, HEK293T cells were transfected with plasmids encoding (i) an sgRNA targeting the endogenous *CCR5* locus or a non-targeting control and (ii) wild-type Cas9 or a Cas9 variant carrying a GR2 domain insertion after the indicated Cas9 residue. Samples were incubated at 37°C or 40°C and cortisol or DMSO were added 2 h post-transfection. InDel frequencies were assessed via T7EI assay after 72 h. Bars indicate InDel frequencies from a single experiment, calculated on the basis of the corresponding gel images shown below. **b**, Indel quantification of the samples corresponding to Fig. 4f by T7EI assay. Data represent the mean +/- SD, n = 3 independent experiments. Representative agarose gel images are shown below each graph. nt, non-targeting; tar., targeting.

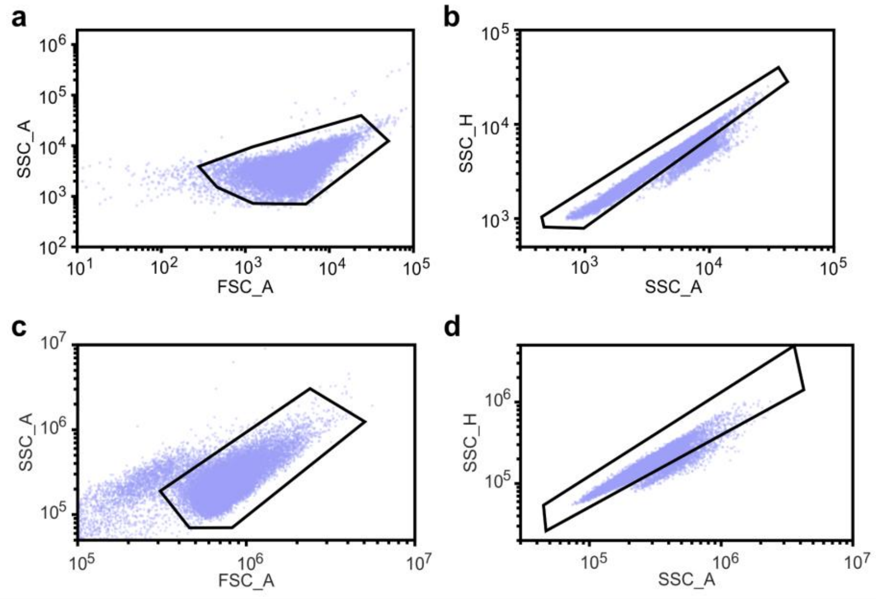

Supplementary Figure 15 | Gating strategy. a-d, *E. coli* (**a-b**) or HEK293T cells (**c-d**) were gated based on a forward vs. side scatter area plots (**a**, **c**). Subsequently, single cells were gated within the SSC height and SSC area channels (**b**, **d**).

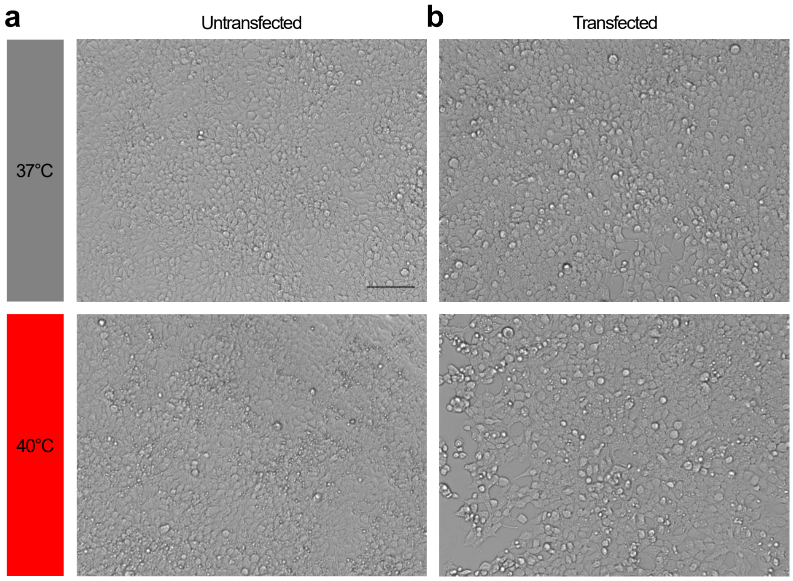

Supplementary Figure 16 | Cell growth at different temperatures. a,b, Untransfected (**a**) or transiently transfected (**b**) HEK293T cells were incubated for 72 hours at the indicated temperatures before microscopy images were acquired. Scale bar indicates 100 μM.

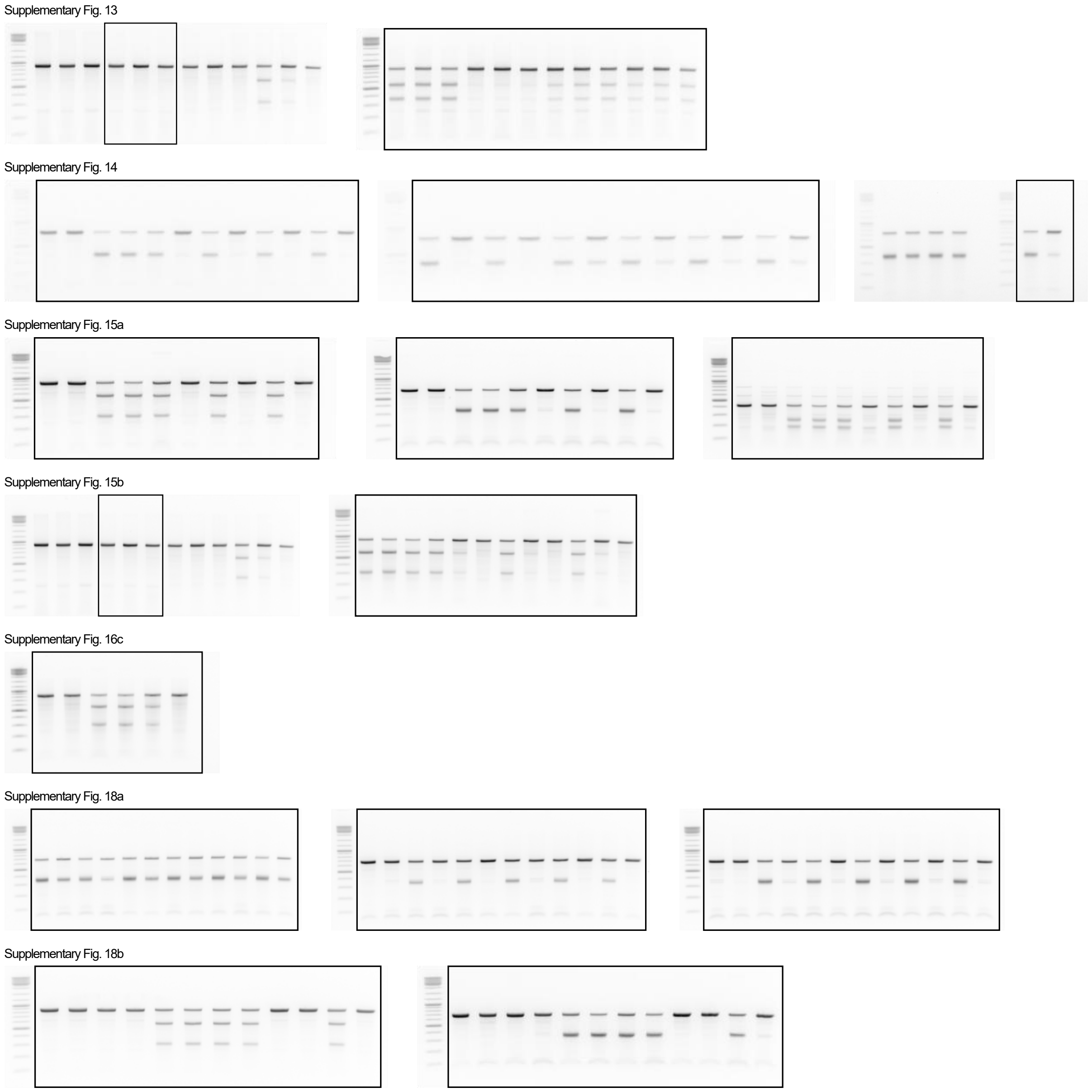

Supplementary Figure 17 | T7 gel images with ladder. Gene Ruler DNA Ladder Mix (Thermo Fisher) was used as a ladder.

Supplementary Table 1 | List of plasmids used in this study. Insert domains are flanked by SG linkers on both sides, unless otherwise indicated. CMV, cytomegalovirus; eGFP, enhanced green fluorescent protein; pConst., constitutive promoter.

#	Name	Description, sequential order	Source
**1**	RFP reporter for AraC	BAD promoter, mRFP1, LVA degradation tag	[2]
**2**	AraC	TRC promoter, AraC	[2]
**3**	TVMV	TRC promoter, TVMV (negative control)	[2]
**4**	BLA	β-lactamase expression cassette	[3]
**5**	BLA_CAT	β-lactamase expression cassette, chloramphenicol acetyltransferase expression cassette	[3]
**6**	BLA_CAT_GSGSG_RFP	β-lactamase expression cassette, chloramphenicol acetyltransferase expression cassette fused to RFP reporter via a GSGSG linker	This work
**7**	Inducible RFP reporter	Arac, pBAD promoter, mRFP1	[2]
**8**	Inducible RFP-LOVdeg	Arac, pBAD promoter, mRFP1 fused to LOVdegron variant	This study
**9**	CRISPRa-RFP-reporter	dCas9-SoxS inducible mRFP	[4]
**10**	dspyCas9_MS2_SoxS_non-targeting	pConst, dSpyCas9; J23107 promoter, MCP fused to SoxS via 5xGS linker; J23119 promoter, non-targeting sgRNA with a MS2 stem loop incorporated into its scaffold	[4]
**11**	dspyCas9_MS2_SoxS_targeting	pConst, dSpyCas9; J23107 promoter, MCP fused to SoxS via 5xGS linker; J23119 promoter, construct #8-targeting sgRNA with a MS2 stem loop incorporated into its scaffold	[4]
**12**	DNA stuffer plasmid	pBluescript sk-	Invitrogen
**13**	H2B-EGFP	CMV promoter, H2B fused to EGFP	[5]
**14**	NLS-mCherry-AsLOV2	$cMyc^{P1A}$ NLS, mCherry-AsLOV2-WT	[6]
**15**	NLS-mCherry-LEXY	$cMyc^{P1A}$ NLS, mCherry-AsLOV2-NES21	[6]
**16**	NLS-mCherry-LEXY-C450A-K488N	$cMyc^{P1A}$ NLS, mCherry-AsLOV2-C450A-K488N-NES21	This work
**17**	NLS-mCherry-NES16-C450A	$cMyc^{P1A}$ NLS, mCherry-AsLOV2-C450A-NES16	This work
**18**	AcrIIA5	CMV promoter, wild-type AcrIIA5 from Streptococcus thermophilus, bGHpA	[7]
**19**	SauCas9_sgRNA-scaffold	CMV promoter, NLS, SauCas9, NLS, 3xHA, bGHpA; U6 promoter, sgRNA scaffold	[8]
**20**	SauCas9_EMX1-sgRNA	CMV promoter, NLS, SauCas9, NLS, 3xHA, bGHpA; U6 promoter, EMX1-targeting sgRNA	[9]
**21**	SauCas9_GRIN2B-sgRNA	CMV promoter, NLS, SauCas9, NLS, 3xHA, bGHpA; U6 promoter, GRIN2B-targeting sgRNA	[9]
**22**	SpyCas9	CMV promoter, 3xFlag, NLS, SpCas9, NLS	[10]
**23**	CFTR-sgRNA (SpyCas9)	U6 promoter, CFTR-targeting sgRNA, RSV GFP	[10]
**24**	CCR5-sgRNA (SpyCas9)	U6 promoter, CCR51-targeting sgRNA, RSV GFP	[11]
**25**	AAVS1-sgRNA (SpyCas9)	U6 promoter, AAVS1-targeting sgRNA, RSV GFP	This work
**26**	dSpyCas9-VPR	Ef1a promoter, NLS, dSpCas9, NLS, VPR	[12]
**27**	mCherry-CRISPRa reporter	TetO repeats, mCherry-MODC, CMV promoter, EGFP-MODC	This work
**28**	TetO-sgRNA (SpyCas9)	U6 promoter, TetO-targeting sgRNA	[13]
**29**	sspB-VPR	CMV promoter, MGSG, NLS, sspB nano fused to VPR via (GGGGS)2	This work
**30**	d*Spy*Cas9-LOV2-ssrA	CMV promoter, dSpyCas9 fused to LOV-ssrA fusion via GGSGG linker	This work
**31**	dCas9-oLID	CMV promoter, dSpyCas9 fused to oLID via GGSGG linker	This work
**32**	dCas9-iLID	CMV promoter, dSpyCas9 fused to iLID via GGSGG linker	This work

Supplementary Table 2 | Amino acid sequences of the domains and proteins used in this study. Blue: linker sequences; orange: affinity tag; green: nuclear localization sequence.

Protein / Domain	Amino acid sequence
***As*LOV2**	LATTLERIEKNFVITDPRLPDNPIIFASDSFLQLTEYSREEILGRNCRFLQGPETDRATVRKIRD<br>AIDNQTEVTVQLINYTKSGKKFWNLFHLQPMRDQKGDVQYFIGVQLDGTEHVRDAAEREGV<br>MLIKKTAENIDEAAK
**AraC**	MSAEAQNDPLLPGYSFNAHLVAGLTPIEANGYLDFFIDRPLGMKGYILNLTIRGQGVVKNQGR<br>EFVCRPGDILLFPPGEIHHYGRHPEAREWYHQWVYFRPRAYWHEWLNWPSIFANTGFFRP<br>DEAHQPHFSDLFGQIINAGQGEGRYSELLAINLLEQLLLRRMEAINESLHPPMDNRVREACQ<br>YISDHLADSNFDIASVAQHVCLSPSRLSHLFRQQLGISVLSWREDQRISQAKLLLSTTRMPIAT<br>VGRNVGFDDQLYFSRVFKKCTGASPSEFRAGCEEKVNDVAVKLSGHHHHHH
**Chloramphenicol acetyltransferase**	MEKKITGYTTVDISQWHRKEHFEAFQSVAQCTYNQTVQLDITAFLKTVKKNKHKFYPAFIHILA<br>RLMNAHPEFRMAMKDGELVIWDSVHPCYTVFHEQTETFSSLLSEYHDDFRQFLHIYSQDVA<br>CYGENLAYFPKGFIENMFFVSANPWVSFTSFDLNVANMDNFFAPVFTMGKYYTQGDKVLMP<br>LAIQVHHAVCDGFHVGRMLNELQQYCDEWQGGA
**RFP-LOVdeg**	MASSEDVIKEFMRFKVRMEGSVNGHEFEIEGEGEGRPYEGTQTAKLKVTKGGPLPFAWDIL<br>SPQFQYGSKAYVKHPADIPDYLKLSFPEGFKWERVMNFEDGGVVTVTQDSSLQDGEFIYKV<br>KLRGTNFPSDGPVMQKKTMGWEASTERMYPEDGALKGEIKMRLKLKDGGHYDAEVKTTYM<br>AKKPVQLPGAYKTDIKLDITSHNEDYTIVEQYERAEGRHSTGASGLATTLERIEKNFVITDPRL<br>PDNPIIFASDSFLQLTEYSREEILGRNARFLQGPETDRATVRKIRDAIDNQTEVTVQLINYTKS<br>GKKFWNLFHLQPMRDQKGDVQYFIGVQLDGTEHVRDAAEREGVMLIKKTAENIDEAA
**MCP-SoxS**	MGPASNFTQFVLVDNGGTGDVTVAPSNFANGIAEWISSNSRSQAYKVTCSVRQSSAQNRKY<br>TIKVEVPKGAWRSYLNMELTIPIFATNSDCELIVKAMQGLLKDGNPIPSAIAANSGIYGGGGSM<br>SHQKIIQDLIAWIDEHIDQPLNIDVVAKKSGYSKWYLQRMFRTVTHQTLGDYIRQRRLLLAAVE<br>LRTTERPIFDIAMDLGYVSQQTFSRVFARQFDRTPADYRHRL
**mCherry-LEXY**	MAAAKRVKLDVSKGEEDNMAIIKEFMRFKVHMEGSVNGHEFEIEGEGEGRPYEGTQTAKLK<br>VTKGGPLPFAWDILSPQFMYGSKAYVKHPADIPDYLKLSFPEGFKWERVMNFEDGGVVTVT<br>QDSSLQDGEFIYKVKLRGTNFPSDGPVMQKKTMGWEASSERMYPEDGALKGEIKQRLKLKD<br>GGHYDAEVKTTYKAKKPVQLPGAYNVNIKLDITSHNEDYTIVEQYERAEGRHSTGGMDELYK<br>GGSGGSGGSLATTLERIEKNFVITDPRLPDNPIIFASDSFLQLTEYSREEILGRNCRFLQGPET<br>DRATVRKIRDAIDNQTEVTVQLINYTKSGKKFWNLFHLQPMRDQKGDVQYFIGVQLDGTEHV<br>RDAAEREGVMLIKKTAENIDELLKELADLNLD
***Spy*Cas9**	MDYKDHDGDYKDHDIDYKDDDDKMAPKKKRKVGIHGVPAADKKYSIGLDIGTNSVGWAVITD<br>EYKVPSKKFKVLGNTDRHSIKKNLIGALLFDSGETAEATRLKRTARRRYTRRKNRICYLQEIFS<br>NEMAKVDDSFFHRLEESFLVEEDKKHERHPIFGNIVDEVAYHEKYPTIYHLRKKLVDSTDKAD<br>LRLIYLALAHMIKFRGHFLIEGDLNPDNSDVDKLFIQLVQTYNQLFEENPINASGVDAKAILSAR<br>LSKSRRLENLIAQLPGEKKNGLFGNLIALSLGLTPNFKSNFDLAEDAKLQLSKDTYDDDLDNLL<br>AQIGDQYADLFLAAKNLSDAILLSDILRVNTEITKAPLSASMIKRYDEHHQDLTLLKALVRQQLP<br>EKYKEIFFDQSKNGYAGYIDGGASQEEFYKFIKPILEKMDGTEELLVKLNREDLLRKQRTFDN<br>GSIPHQIHLGELHAILRRQEDFYPFLKDNREKIEKILTFRIPYYVGPLARGNSRFAWMTRKSEE<br>TITPWNFEEVVDKGASAQSFIERMTNFDKNLPNEKVLPKHSLLYEYFTVYNELTKVKYVTEG<br>MRKPAFLSGEQKKAIVDLLFKTNRKVTVKQLKEDYFKKIECFDSVEISGVEDRFNASLGTYHD<br>LLKIIKDKDFLDNEENEDILEDIVLTLTLFEDREMIEERLKTYAHLFDDKVMKQLKRRRYTGWG<br>RLSRKLINGIRDKQSGKTILDFLKSDGFANRNFMQLIHDDSLTFKEDIQKAQVSGQGDSLHEHI<br>ANLAGSPAIKKGILQTVKVVDELVKVMGRHKPENIVIEMARENQTTQKGQKNSRERMKRIEE<br>GIKELGSQILKEHPVENTQLQNEKLYLYYLQNGRDMYVDQELDINRLSDYDVDHIVPQSFLKD<br>DSIDNKVLTRSDKNRGKSDNVPSEEVVKKMKNYWRQLLNAKLITQRKFDNLTKAERGGLSEL<br>DKAGFIKRQLVETRQITKHVAQILDSRMNTKYDENDKLIREVKVITLKSKLVSDFRKDFQFYKV<br>REINNYHHAHDAYLNAVVGTALIKKYPKLESEFVYGDYKVYDVRKMIAKSEQEIGKATAKYFF<br>YSNIMNFFKTEITLANGEIRKRPLIETNGETGEIVWDKGRDFATVRKVLSMPQVNIVKKTEVQT<br>GGFSKESILPKRNSDKLIARKKDWDPKKYGGFDSPTVAYSVLVVAKVEKGKSKKLKSVKELL<br>GITIMERSSFEKNPIDFLEAKGYKEVKKDLIIKLPKYSLFELENGRKRMLASAGELQKGNELAL<br>PSKYVNFLYLASHYEKLKGSPEDNEQKQLFVEQHKHYLDEIIEQISEFSKRVILADANLDKVLS

	AYNKHRDKPIREQAENIIHLFTLTNLGAPAAFKYFDTTIDRKRYTSTKEVLDATLIHQSITGLYE<br>TRIDLSQLGGDKRPAATKKAGQAKKKKEF
***Sau*Cas9**	MAPKKKRKVGIHGVPAAKRNYILGLDIGITSVGYGIIDYETRDVIDAGVRLFKEANVENNEGRR<br>SKRGARRLKRRRRHRIQRVKKLLFDYNLLTDHSELSGINPYEARVKGLSQKLSEEEFSAALLH<br>LAKRRGVHNVNEVEEDTGNELSTKEQISRNSKALEEKYVAELQLERLKKDGEVRGSINRFKT<br>SDYVKEAKQLLKVQKAYHQLDQSFIDTYIDLLETRRTYYEGPGEGSPFGWKDIKEWYEMLM<br>GHCTYFPEELRSVKYAYNADLYNALNDLNNLVITRDENEKLEYYEKFQIIENVFKQKKKPTLK<br>QIAKEILVNEEDIKGYRVTSTGKPEFTNLKVYHDIKDITARKEIIENAELLDQIAKILTIYQSSEDIQ<br>EELTNLNSELTQEEIEQISNLKGYTGTHNLSLKAINLILDELWHTNDNQIAIFNRLKLVPKKVDL<br>SQQKEIPTTLVDDFILSPVVKRSFIQSIKVINAIIKKYGLPNDIIIELAREKNSKDAQKMINEMQKR<br>NRQTNERIEEIIRTTGKENAKYLIEKIKLHDMQEGKCLYSLEAIPLEDLLNNPFNYEVDHIIPRSV<br>SFDNSFNNKVLVKQEENSKKGNRTPFQYLSSSDSKISYETFKKHILNLAKGKGRISKTKKEYL<br>LEERDINRFSVQKDFINRNLVDTRYATRGLMNLLRSYFRVNNLDVKVKSINGGFTSFLRRKW<br>KFKKERNKGYKHHAEDALIIANADFIFKEWKKLDKAKKVMENQMFEEKQAESMPEIETEQEY<br>KEIFITPHQIKHIKDFKDYKYSHRVDKKPNRELINDTLYSTRKDDKGNTLIVNNLNGLYDKDND<br>KLKKLINKSPEKLLMYHHDPQTYQKLKLIMEQYGDEKNPLYKYYEETGNYLTKYSKKDNGPVI<br>KKIKYYGNKLNAHLDITDDYPNSRNKVVKLSLKPYRFDVYLDNGVYKFVTVKNLDVIKKENYY<br>EVNSKCYEEAKKLKKISNQAEFIASFYNNDLIKINGELYRVIGVNNDLLNRIEVNMIDITYREYLE<br>NMNDKRPPRIIKTIASKTQSIKKYSTDILGNLYEVKSKKHPQIIKKGKRPAATKKAGQAKKKKG<br>SYPYDVPDYAYPYDVPDYAYPYDVPDYA
**cpGR2**	SGNSSQNWQRFYQLTKLLDSMHEMVGGLLQFCFYTFVNKSLSVEFPEMLAEIISNQLPKFNA<br>GSVKPLLFHQKGGGSGGSGGSGGSGGSGLISLLEVIEPEVLYSGYDSTLPDTSTRLMSTLNR<br>LGGRQVVSAVKWAKALPGFRNLHLDDQMTLLQYSWMSLMAFSLGWRSYKQSNGNMLCFA<br>PDLVINEERMQLPYMYDQCQQMLKISSEFVRLQVSYDEYLCMKVLLLLSTVPKDGLKSQAVF<br>DEIRMTYIKELGKAIVKREGGS
**AcrIIA5**	MAYGKSRYNSYRKRNFSISDNQRREYAKKMKELEQAFENLDGWYLSSMKDSAYKDFGKYEI<br>RLSNHSADNRYHDLENGRLIVNVKASKLNFVDIIENKLGKIIEKIDTLDLDKYRFINATKLERDIK<br>CYYKGYKTKKDVI
**sspB nano**	SSPKRPKLLREYYDWLVDNSFTPYLVVDATYLGVNVPVEYVKDGQIVLNLSASATGNLQLTN<br>DFIQFNARFKGVSRELYIPMGAALAIYARENGDGVMFEPEEIYDELNIG
**LOV-ssrA fusion**	LATTLERIEKNFVITDPRLPDNPIIFASDSFLQLTEYSREEILGRNCRFLQGPETDRATVRKIRD<br>AIDNQTEVTVQLINYTKSGKKFWNLFHLQPMRDQKGDVQYFIGVQLDGTEHVRDAAEREGV<br>MLIKKTAENIDEAANDENY
**oLID**	LATTLERIEKNFVITDPRLPDNPIIFASDSFLQLTEYSREEILGRNCRFLQGPETDRATVRKIRD<br>AIDNQTEVTVQLINYTKSGKKFWNLFHLQPMRDQKGDVQYFIGVQLDGTEHVRDAAEREAV<br>MLIKKTAEEIDEAANDENYF
**iLID**	LATTLERIEKNFVITDPRLPDNPIIFASDSFLQLTEYSREEILGRNCRFLQGPETDRATVRKIRD<br>AIDNQTEVTVQLINYTKSGKKFWNVFHLQPMRDYKGDVQYFIGVQLDGTERLHGAAEREAV<br>CLIKKTAFQIAEAANDENYF

Supplementary Table 3 | Hybrid proteins used in this study. The insertion site corresponds to the residue in the effector protein preceding the insert domain. The sequences of the two linkers flanking the insert domain are shown.

#	Name	Protein	Insertion site	Linker	Mutations
**1**	AraC-S170-LOV	AraC	S170	SG-GS	-
**2**					C450A
**3**					C450A/E409C
**4**					C450A/E409R
**5**					C450A/E409V
**6**					C450A/D432V
**7**					C450A/R448S
**8**					C450A/D456L
**9**					C450A/D459E
**10**					C450A/R460H
**11**					C450A/R464H
**12**					C450A/S486C
**13**					C450A/K488N
**14**					C450A/R464H/L531F
**15**					C450A/P423R/R460H/D515V
**16**					C450A/D432V/K488N
**17**					T406A/T407A
**18**					I532A
**19**					N538E
**20**					G528A/N538E
**21**	CAT-K136-LOV	CAT	K136	-	-
**22**				G-G	
**23**				SG-GS	
**24**				GP-PG	
**25**				PG-GP	
**26**				GPG-GPG	
**27**				K136	C450A
**28**					C450A/E409C
**29**					C450A/E409V
**30**					C450A/D432V
**31**					C450A/K488N
**32**	CAT-K136-LOV-GSGSG-RFP	CAT-RFP	K136	K136	C450A
**33**					C450A/E409C
**34**					C450A/E409V
**35**					C450A/D432V
**36**					C450A/K488N
**37**	mRFP-LOV	mRFP	A225	SG	C450A, EAAKGS
**38**					C450A, EAAKGS, E409V
**39**					C450A, EAAKGS, L493V
**40**					C450A, LAAKGS, E409V
**41**				SSGSG	C450A, EAA
**42**					C450A, LAA
**43**	MCP-SoxS	MCP	N27	GS-SG	C450A
**44**			N27		C450A, K488N
**45**			A56		C450A
**46**			A97		
**47**		MCP-SoxS	linker in between	G-S	
**48**	AcrIIA5-E76-LOV	AcrIIA5	E76	-	C450A
**49**					C450A/K488N
**50**	SpyCas9-E566-LOV	SpyCas9	E566	SG-GS	-
**51**					C450A
**52**					C450A/D432V
**53**					C450A/P456L
**54**					C450A/D459E
**55**					C450A/K488N
**56**	SpyCas9-T1048-LOV	SpyCas9	T1048	SG-GS	-
**57**					C450A
**58**					C450A/P456L
**59**					C450A/D459E
**60**					C450A/D432V
**61**					C450A/K488N
**62**	SpyCas9-E566-cpGR2	SpyCas9	E566	SG-GS	-
**63**	SpyCas9-T1048-cpGR2	SpyCas9	T1048	SG-GS	-
**64**	dSpyCas9-E566-LOV-VPR	dSpyCas9	E566	SG-GS	-
**65**					C450A
**66**					C450A/K488N

Supplementary Table 4 | Genomic target sites of the sgRNAs used in this study. Spacer sequences are marked in bold, PAM motifs are underlined.

Cas protein	Target locus	Sequence 5'-3'	Source
*Spy*Cas9	*CCR5*	**TGACATCAATTATTATACAT**CGG	[11]
	CFTR	**AATGGTGCCAGGCATAATCC**AGG	[10]
	AAVS1	**GGGGCCACTAGGGACAGGAT**TGG	[14]
	TetO	**TCTCTATCACTGATAGGGAG**TGG	[13]
*Sau*Cas9	*EMX1*	**GGCCTCCCCAAAGCCTGGCCA**GGGAGT	[9]
	GRIN2B	**GAGAGTAGGCTGGTAGATGGAG**TTGGGT	[9]

Supplementary Table 5 | Primers used for amplification of genomic loci.

Gene	Orientation	Primer sequence 5'-3'
EMX1	Forward	GGGCCTGAGTCCGAGCAGAAG
	Reverse	CAAAAGGGAGATTGGAGACACG
GRIN2B	Forward	CAGGACGGCCAACACCAAC
	Reverse	GTGTATGCATACTCGCATGGC
AAVS1	Forward	GACAGCATGTTTGCTGCCTC
	Reverse	CTCCCTCCCAGGATCCTCTC
CCR5	Forward	GAAGGAAAAACAGGTCAGAG
	Reverse	CATTGCTTGGCCAAAAAGAGAG
CFTR	Forward	GACTAACCGATTGAATATGGAG
	Reverse	ATACACTTCTGCTTAGGATGA

Supplementary references

1. Niopek, D., Wehler, P., Roensch, J., Eils, R. & Di Ventura, B. Optogenetic control of nuclear protein export. *Nat. Commun.* **7**, 1–9 (2016).
2. Mathony, J., Aschenbrenner, S., Becker, P. & Niopek, D. Dissecting the Determinants of Domain Insertion Tolerance and Allostery in Proteins. *Adv. Sci.* **10**, 2303496 (2023).
3. Wolf, B. *et al.* Rational engineering of allosteric protein switches by in silico prediction of domain insertion sites. *Nat. Methods* **22**, 1698–1706 (2025).
4. Fontana, J. *et al.* Effective CRISPRa-mediated control of gene expression in bacteria must overcome strict target site requirements. *Nat. Commun.* **11**, 1618 (2020).
5. Kanda, T., Sullivan, K. F. & Wahl, G. M. Histone–GFP fusion protein enables sensitive analysis of chromosome dynamics in living mammalian cells. *Curr. Biol.* **8**, 377–385 (1998).
6. Niopek, D., Wehler, P., Roensch, J., Eils, R. & Ventura, B. D. Optogenetic control of nuclear protein export. *Nat. Commun.* 1–9 (2016) doi:10.1038/ncomms10624.
7. Brenker, L. *et al.* A versatile anti-CRISPR platform for opto- and chemogenetic control of CRISPR-Cas9 and Cas12 across a wide range of orthologs. *Nucleic Acids Res.* **53**, gkaf752 (2025).
8. Ran, F. A. *et al.* In vivo genome editing using Staphylococcus aureus Cas9. *Nature* **520**, 186–91 (2015).
9. Mathony, J. *et al.* Computational design of anti-CRISPR proteins with improved inhibition potency. *Nat. Chem. Biol.* **16**, 725–730 (2020).
10. Bubeck, F. *et al.* Engineered anti-CRISPR proteins for optogenetic control of CRISPR–Cas9. *Nat. Methods* **15**, 924–927 (2018).
11. Nihongaki, Y., Kawano, F., Nakajima, T. & Sato, M. Photoactivatable CRISPR-Cas9 for optogenetic genome editing. *Nat. Biotechnol.* **33**, 755–760 (2015).
12. Muench, P. *et al.* A modular toolbox for the optogenetic deactivation of transcription. *Nucleic Acids Res.* gkae1237 (2024) doi:10.1093/nar/gkae1237.
13. Nihongaki, Y., Yamamoto, S., Kawano, F., Suzuki, H. & Sato, M. CRISPR-Cas9-based Photoactivatable Transcription System. *Chem. Biol.* **22**, 169–174 (2015).
14. Mali, P. *et al.* RNA-guided human genome engineering via Cas9. *Science* **339**, 823–826 (2013).
