# Supplementary material for: Modular engineering of thermoresponsive allosteric proteins
Source: Nat Chem Biol. 2026 Feb 12;22(5):751–8. doi: 10.1038/s41589-026-02151-y (PMC13128441; doi:10.1038/s41589-026-02151-y)
Supplement: Supplementary file 2 — Reporting Summary [file 41589_2026_2151_MOESM2_ESM.pdf]

nature portfolio

Corresponding author(s): Dr. Jan Mathony, Prof. Dr. Dominik Niopek

Last updated by author(s): 2026/01/12

Reporting Summary

Nature Portfolio wishes to improve the reproducibility of the work that we publish. This form provides structure for consistency and transparency in reporting. For further information on Nature Portfolio policies, see our Editorial Policies and the Editorial Policy Checklist.

Statistics

For all statistical analyses, confirm that the following items are present in the figure legend, table legend, main text, or Methods section.

n/a	Confirmed	
☐	☒	The exact sample size (*n*) for each experimental group/condition, given as a discrete number and unit of measurement
☐	☒	A statement on whether measurements were taken from distinct samples or whether the same sample was measured repeatedly
☐	☒	The statistical test(s) used AND whether they are one- or two-sided<br>*Only common tests should be described solely by name; describe more complex techniques in the Methods section.*
☒	☐	A description of all covariates tested
☐	☒	A description of any assumptions or corrections, such as tests of normality and adjustment for multiple comparisons
☐	☒	A full description of the statistical parameters including central tendency (e.g. means) or other basic estimates (e.g. regression coefficient) AND variation (e.g. standard deviation) or associated estimates of uncertainty (e.g. confidence intervals)
☐	☒	For null hypothesis testing, the test statistic (e.g. *F*, *t*, *r*) with confidence intervals, effect sizes, degrees of freedom and *P* value noted<br>*Give P values as exact values whenever suitable.*
☒	☐	For Bayesian analysis, information on the choice of priors and Markov chain Monte Carlo settings
☒	☐	For hierarchical and complex designs, identification of the appropriate level for tests and full reporting of outcomes
☒	☐	Estimates of effect sizes (e.g. Cohen's *d*, Pearson's *r*), indicating how they were calculated

Our web collection on statistics for biologists contains articles on many of the points above.

Software and code

Policy information about availability of computer code

Data collection	Luciferase data, fluorescence, absorbance and optical density data were obtained using the i-control software (Tecan, version 2.0). Light microscopy images were acquired and processed with the Keyence BZ-II Viewer (Keyence, version 1.01) and BZ-II Analyzer software (Keyence, version 1.01). Fluorescence microscopy images were acquired with a Nikon TIRF microscope. Western blot images were captured with a LAS 3000 Imaging System, Fuji. Flow cytometry was performed using a Cytoflex S cytometer using Cytexpert 2.5.0.77.
Data analysis	Analysis of NGS data was performed using and CRISPResso2 2.3.1. Fluorescence microscopy images were analyzed using Fiji 2.16.0. Cytometry data was analyzed using the Cytoflow v1.3.0 software package.

For manuscripts utilizing custom algorithms or software that are central to the research but not yet described in published literature, software must be made available to editors and reviewers. We strongly encourage code deposition in a community repository (e.g. GitHub). See the Nature Portfolio guidelines for submitting code & software for further information.

Data

Policy information about availability of data

All manuscripts must include a data availability statement. This statement should provide the following information, where applicable:
- Accession codes, unique identifiers, or web links for publicly available datasets
- A description of any restrictions on data availability
- For clinical datasets or third party data, please ensure that the statement adheres to our policy

Additional information, including relevant amino acid sequences, targeted genomic loci and PCR primers for InDel quantification are provided in the Supplementary Information. Genbank files of the DNA constructs are provided as Supplementary Data files. Important constructs will be shared on Addgene (Addgene ID: xxxx, xxxx). Additional data will be shared upon reasonable request.

Research involving human participants, their data, or biological material

Policy information about studies with human participants or human data. See also policy information about sex, gender (identity/presentation), and sexual orientation and race, ethnicity and racism.

Reporting on sex and gender	n/a
Reporting on race, ethnicity, or other socially relevant groupings	n/a
Population characteristics	n/a
Recruitment	n/a
Ethics oversight	n/a

Note that full information on the approval of the study protocol must also be provided in the manuscript.

Field-specific reporting

Please select the one below that is the best fit for your research. If you are not sure, read the appropriate sections before making your selection.

☒ Life sciences ☐ Behavioural & social sciences ☐ Ecological, evolutionary & environmental sciences

For a reference copy of the document with all sections, see nature.com/documents/nr-reporting-summary-flat.pdf

Life sciences study design

All studies must disclose on these points even when the disclosure is negative.

Sample size	Sample sizes were chosen on the basis of an initial pilot experiment and further based on similar experiments reported in previous publications.
Data exclusions	No experimental data were excluded from the analysis.
Replication	All attempts for replication were successful. The number of replicates performed is indicated in each figure legend, where applicable.
Randomization	No randomization was used, as samples and controls were treated side-by-side using the identical protocols and workflows for analysis. Also, the study did not involve human or animal subjects.
Blinding	No blinding was used, as the majority of data was automatically collected by machines and analyzed by standard workflows.

Reporting for specific materials, systems and methods

We require information from authors about some types of materials, experimental systems and methods used in many studies. Here, indicate whether each material, system or method listed is relevant to your study. If you are not sure if a list item applies to your research, read the appropriate section before selecting a response.

Materials & experimental systems

n/a	Involved in the study
☐	☒ Antibodies
☐	☒ Eukaryotic cell lines
☒	☐ Palaeontology and archaeology
☒	☐ Animals and other organisms
☒	☐ Clinical data
☒	☐ Dual use research of concern
☒	☐ Plants

Methods

n/a	Involved in the study
☒	☐ ChIP-seq
☐	☒ Flow cytometry
☒	☐ MRI-based neuroimaging

Antibodies

Antibodies used: mouse anti-His IgG primary antibody (Proteintech, 66005-1-Ig, 1:50,000); goat anti-Mouse IgG secondary antibody (Jackson Immuno Research, 115-035-068, 1:10,000)

Validation: Both antibodies have been validated for Western Blot in various dilutions.

Eukaryotic cell lines

Policy information about cell lines and Sex and Gender in Research

Cell line source(s): HEK 293T cells were kindly provided by Ellen Wiedtke (Heidelberg University clinics and BioQuant, Heidelberg, Germany).

Authentication: The cell line was authenticated via Single Nucleotide Polymorphism Profiling using a commercially available service (Multiplexion, Heidelberg, Germany) in March 2025.

Mycoplasma contamination: The cell line was tested negative for mycoplasma contamination via a commercially available service (Multiplexion, Heidelberg, Germany) prior to usage.

Commonly misidentified lines (See ICLAC register): HEK (293T) is a standard cell line widely used for transient transfection and CRISPR/Cas9 experiments.

Plants

Seed stocks: *Report on the source of all seed stocks or other plant material used. If applicable, state the seed stock centre and catalogue number. If plant specimens were collected from the field, describe the collection location, date and sampling procedures.*

Novel plant genotypes: *Describe the methods by which all novel plant genotypes were produced. This includes those generated by transgenic approaches, gene editing, chemical/radiation-based mutagenesis and hybridization. For transgenic lines, describe the transformation method, the number of independent lines analyzed and the generation upon which experiments were performed. For gene-edited lines, describe the editor used, the endogenous sequence targeted for editing, the targeting guide RNA sequence (if applicable) and how the editor was applied.*

Authentication: *Describe any authentication procedures for each seed stock used or novel genotype generated. Describe any experiments used to assess the effect of a mutation and, where applicable, how potential secondary effects (e.g. second site T-DNA insertions, mosiacism, off-target gene editing) were examined.*

Flow Cytometry

Plots

Confirm that:

☒ The axis labels state the marker and fluorochrome used (e.g. CD4-FITC).

☒ The axis scales are clearly visible. Include numbers along axes only for bottom left plot of group (a 'group' is an analysis of identical markers).

☐ All plots are contour plots with outliers or pseudocolor plots.

☒ A numerical value for number of cells or percentage (with statistics) is provided.

Methodology

Sample preparation: E. coli were diluted in PBS prior to measurement. HEK293T cells were washed with 1x phosphate-buffered saline (PBS), trypsinized and resuspended in 1x DMEM without phenol red supplemented with 10% (v/v) fetal bovine serum, 2 mM L-glutamine, 100 U/mL penicillin, and 100 µg/mL streptomycin (all from Thermo Fisher Scientific). The cells were transferred to 2 ml tubes and kept on ice from this stage onward. The samples were washed with 800 µL PBS, resuspended in 250 µL PBS

	and passed through a 0.45 µM cell strainer.
Instrument	Cytoflex S (Beckman Coulter)
Software	CytExpert (Beckman Coulter)
Cell population abundance	20,000 cells
Gating strategy	E. coli or HEK293T cells were gated based on a forward vs. side scatter area plots. Subsequently, single cells were gated within the SSC height and SSC area channels.

☒ Tick this box to confirm that a figure exemplifying the gating strategy is provided in the Supplementary Information.
